# Fecal microbiota transplantation ameliorates radiation-induced lung injury by reshaping gut metabolic homeostasis to activate FAM134B-mediated ER-phagy

**DOI:** 10.1371/journal.ppat.1013786

**Published:** 2026-01-21

**Authors:** Xiaoyu Pu, Bohao Liu, Lihua Dong, Meng Yuan, Shunzi Jin, Xin Jiang

**Affiliations:** 1 Jilin Provincial Key Laboratory of Radiation Oncology & Therapy, Department of Radiation Oncology & Therapy, The First Hospital of Jilin University, Changchun, China; 2 Department of Thoracic Surgery, The First Hospital of Jilin University, Changchun, China; 3 National Health Commission Key Laboratory of Radiobiology, School of Public Health, Jilin University, Changchun, China; Universite Paris Descartes Faculte de Medecine, FRANCE

## Abstract

Radiation-induced lung injury (RILI) is a serious complication of thoracic radiotherapy, with limited effective treatment options. This study demonstrates that fecal microbiota transplantation (FMT) confers protection against RILI through modulation of the gut-lung axis. In a total lung irradiation (TLI) mouse model, FMT significantly alleviated pulmonary histopathological injury, inflammatory responses, oxidative stress, and collagen deposition during fibrogenesis. Concurrently, FMT improved intestinal motility, enhanced mucosal barrier integrity, and restored TLI-induced dysbiosis in gut microbiota diversity and community structure. Metabolomic analysis revealed that TLI significantly disrupted the metabolism of unsaturated fatty acids and arachidonic acid (AA), whereas FMT partially restored these metabolic networks. Transcriptomic and ultrastructural analyses indicated that RILI suppressed endoplasmic reticulum (ER) protein processing and induced ER swelling, while FMT promoted protective ER-phagy and facilitated restoration of ER morphology. Integrated multi-omics analysis further identified the AA metabolism as a key component of FMT-mediated protection, with its alterations closely associated with pulmonary tissue repair. Further *in vivo* and *in vitro* experiments demonstrated that AA binds to and activates the nuclear receptor PPARγ, leading to transcriptional upregulation of FAM134B, promoting protective ER-phagy and ameliorating RILI. In summary, this study highlights the bidirectional gut-lung axis as a therapeutic target in RILI progression and intervention, and reveals that FMT confers protection through metabolic remodeling and activation of the PPARγ-FAM134B-mediated ER-phagy pathway, providing a mechanistic basis for potential clinical translation.

## Author summary

RILI is a severe complication of thoracic radiotherapy, yet effective therapies remain limited. We comprehensively delineate the pathogenesis and therapeutic interventions of RILI via the gut-lung axis, emphasizing bidirectional regulation. Thoracic irradiation can compromise intestinal motility and disrupt mucosal barrier integrity via the abscopal effect, whereas FMT restores barrier function and microbial homeostasis, reshapes gut ecology, and enhances pulmonary repair. We define FMT as a systemic intervention that resets metabolic homeostasis and promotes ER adaptation from organismal to organelle levels. Mechanistically, gut‑derived lipid metabiolism, with AA as a key component, activate PPARγ and transcriptionally upregulate FAM134B, thereby enhancing selective ER‑phagy and mitigating RILI. Overall, this study reveals that RILI is not confined to localized pulmonary injury but involves a cross-organ imbalance encompassing metabolic, immune, and organelle adaptive responses. We developed an intervention strategy aimed at restoring gut-lung axis homeostasis to reprogram transcriptional mechanisms governing ER quality control, thereby promoting organ repair and functional reconstruction.

## Introduction

Radiation therapy serves as a foundational approach in the management of various thoracic malignancies. Nevertheless, radiation-induced lung injury (RILI) presents a significant clinical challenge that impedes advancements in local tumor control. RILI is characterized by a biphasic progression: the early phase, known as radiation-induced pneumonitis, manifests as acute lung inflammation subsequent to radiation exposure, typically arising within weeks following radiotherapy. In contrast, the late phase is marked by radiation-induced pulmonary fibrosis, which develops as persistent and irreversible fibrotic changes, commonly occurring 2–6 months post-treatment. Such sequelae profoundly impact patients’ quality of life and overall health outcomes [1]. Currently, the primary clinical intervention for RILI consists of high-dose corticosteroid therapy. however, this approach has demonstrated inadequate efficacy in halting disease progression and may also disrupt electrolyte balance and compromise effector cell survival [2,3].

The gut-lung axis represents a bidirectional communication pathway that links gut and lung health, playing a vital role in the maintenance of respiratory homeostasis [4]. Under normal conditions, bioactive substances produced by the gut microbiota contribute to respiratory system balance through mechanisms such as blood circulation, neural signaling, and immune regulation [5]. These substances are vital for resisting external stress and preserving lung health. However, gut microbiota dysbiosis in the gut can compromise intestinal barrier integrity and affect pulmonary pathophysiology through a “distal effect” mechanism [6]. This impact includes the regulation of immune function, initiation and control of inflammatory responses, and modulation of tissue repair processes [7,8]. Evidence shows that lung diseases can induce gut dysfunction and microbial imbalance, which, through a complex feedback loop, can further exacerbate lung damage [9]. This feedback loop operates not through direct cell contact but via bioactive substances transported through the bloodstream. These include gut microbiota metabolites, signaling molecules from immune and epithelial cells, and extracellular vesicles carrying various signals. These substances circulate throughout the body, regulating systemic inflammatory responses and tissue repair [9,10]. In the context of RILI, the link between gut dysbiosis and pulmonary damage has been further substantiated. Research indicates that whole-lung irradiation can disrupt gut microbiota balance in mice, while the gut microbiota metabolite prostaglandin PGF2α promotes the repair of irradiated lung tissue by activating the MAPK/NF-κB signaling pathway [9]. These findings highlight the complex feedback mechanisms between gut microbiota and distal lung injury and underscore the therapeutic potential of the gut-lung axis in mitigating RILI.

Considering the inherent challenges posed by RILI, which is often deemed “incurable,” the imperative for novel and effective treatment strategies has become increasingly urgent in clinical practice. Accordingly, this study aims to investigate the role of the gut-lung axis in the context of RILI. By employing fecal microbiota transplantation (FMT), we reveal the ameliorative impact of the gut-lung axis on abscopal effects and delineate underlying molecular regulatory mechanisms in RILI mitigation, providing novel insights for targeted therapeutic strategies against RILI.

## Results

### FMT attenuates inflammatory storm and fibrogenesis in radiation-induced lung injury

**Fig 1:**
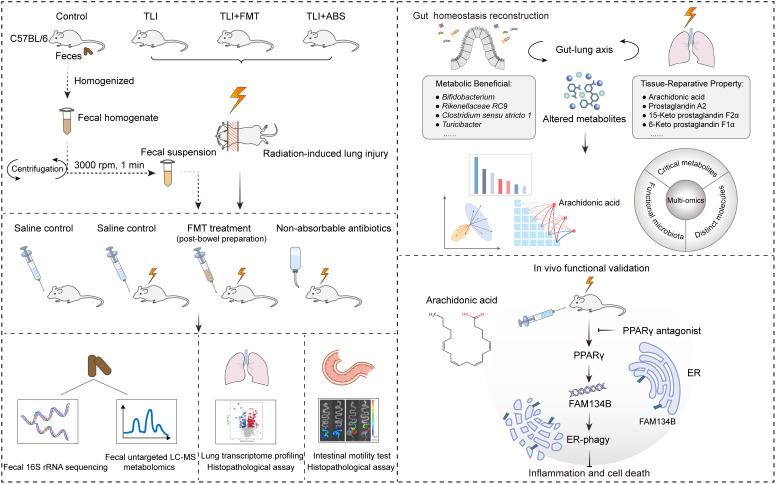
Schematic of fecal microbiota transplantation efficacy assessment in RILI mouse models.

**Fig 2:**
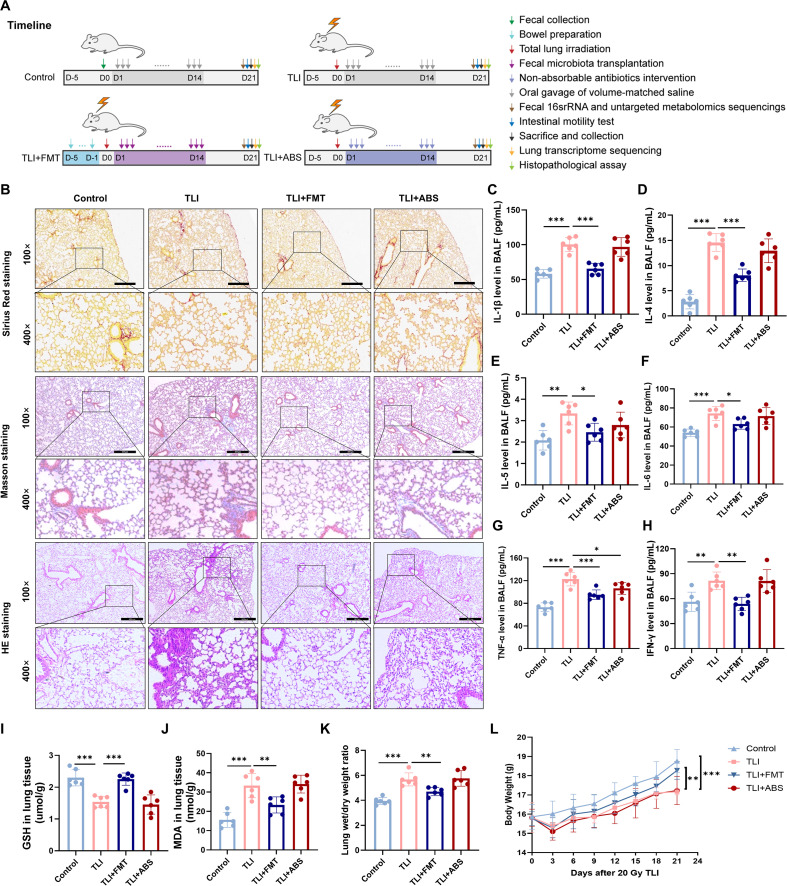
Protective effects of FMT on lung tissue morphology and inflammatory response in RILI mice. **(A)** The timeline of experimental interventions and sampling of this study. **(B)** Representative Sirius Red, Masson’s trichrome, and H&E staining of lung tissues (100× and 400×). **(C-H)** BALF levels of IL-1β, IL-4, IL-5, IL-6, TNF-α and IFN-γ quantified by ELISA. **(I-J)** GSH and MDA levels of lung tissue. **(K)** Lung wet/dry weight ratio across groups. **(L)** The change of body weight of each experimental mice after 20 Gy TLI. n = 6 per group. Data are presented as mean ± SD. Statistical comparisons were performed by one-way ANOVA with Tukey’s post hoc test (C-K) or two-way ANOVA with Tukey’s post hoc test **(L)**. * *p* < 0.05, ** *p* < 0.01, *** *p* < 0.001.

In this study, we established total lung irradiation (TLI) mouse models to systematically investigate the therapeutic potential of FMT in mitigating pulmonary inflammation, tissue damage and collagen deposition. Following the experimental schematic and timeline delineated in Figs 1 and 2A, we systematically characterized pathological manifestations in RILI mice. Our experiments revealed significant changes in lung tissue morphology, bronchoalveolar lavage fluid (BALF), and serum inflammatory markers in RILI mice. Masson staining indicated radiation-induced collagen deposition in lung tissues, and Hematoxylin & Eosin (HE) staining revealed compromised integrity of the alveolar walls and interstitial structures, along with infiltration of inflammatory cells (Fig 2B). Elevated levels of inflammatory factors, including IL-1β, IL-4, IL-5, IL-6, TNF-α, and IFN-γ, were observed in BALF and serum of the TLI group (Figs 2C-2H and S1A-S1E). Concurrently, the TLI group exhibited diminished glutathione (GSH), elevated malondialdehyde (MDA), and increased lung wet/dry weight ratios (Fig 2I-2K), collectively indicating pronounced inflammatory storms and oxidative stress injury. However, FMT treatment significantly reduced proinflammatory cytokines in BALF and serum, and ameliorated oxidative stress and pulmonary edema compared to the TLI group, as evidenced by restored GSH, reduced MDA, and normalized lung wet/dry weight ratios. Additionally, FMT can also prevent the weight loss induced by TLI (Fig 2L). These coordinated improvements indicate FMT-mediated systemic modulation of inflammatory cascades, suppression of cytokine storms, mitigation of oxidative injury, and restoration of pulmonary homeostasis. In contrast, the non-absorbable antibiotics (ABS) intervention exhibited limited effects on inflammation, edema and oxidative injury after TLI.

Conventional FMT protocol includes a broad-spectrum antibiotic preconditioning step to facilitate microbial engraftment. To control for potential confounding effects from the broad-spectrum antibiotic preconditioning and to establish the necessity of this pre-FMT antibiotic regimen, we designed a critical controlled experiment comparing four groups: TLI alone, TLI with antibiotic preconditioning (TLI + pre-Abx), TLI with the conventional FMT protocol, which includes antibiotic preconditioning (TLI + FMT) and TLI with FMT administered without prior antibiotic pretreatment (TLI + FMT/no pre-Abx). Histopathological assessment indicated that, relative to TLI mice, pre‑Abx per se did not ameliorate lung tissue damage or fibrotic remodeling. Moreover, compared with FMT administered following pre‑Abx, FMT delivered without pre‑Abx similarly did not confer measurable protection against TLI‑induced pulmonary injury (S2A-S2C Fig). Similarly, assessment of oxidative stress parameters in lung tissue revealed that pre‑Abx treatment failed to correct the depletion of GSH or the aberrant elevation of MDA, and the TLI + FMT/no pre‑Abx group likewise did not exhibit any protective effect (S2D and S2E Fig). Evaluation of pulmonary edema and body weight changes further demonstrated that neither of these groups conferred improvements comparable to those observed with FMT intervention following pre‑Abx administration, in terms of lung tissue restoration and overall systemic condition in mice (S2F and S2G Fig). Taken together, FMT conferred marked therapeutic efficacy against RILI, mitigating lung tissue damage, fibrogenesis, and inflammatory burden. However, these benefits are dependent upon pre‑Abx to reduce intestinal microbial load and facilitate successful engraftment of the transplanted microbiota, whereas antibiotic pretreatment alone did not exert a clear influence on the course of RILI.

Additionally, FMT treatment attenuated radiation-induced fibrosis in mice, as demonstrated by histopathological assessment at 8 weeks post-irradiation. Masson staining revealed significantly reduced collagen deposition in FMT-treated mice compared to the TLI group, corroborated by attenuated alveolar structural disruption on H&E staining (S3A Fig). Quantitative assessment using the Ashcroft score and lung injury score further confirmed preserved pulmonary architecture (S3B and S2C Figs). Critically, FMT treatment reversed radiation-induced epithelial-to-mesenchymal transition (EMT), normalizing dysregulated EMT markers with elevated *CDH1* expression and suppressed *CDH2*, *ACTA2* and *VIM* expression (S3D Fig). Similar results were observed at the protein level (S3E Fig). These coordinated improvements in EMT dynamics and collagen homeostasis collectively establish FMT’s capacity to mitigate fibrogenesis in RILI.

### Radiation-induced lung injury disrupts gut microbiota in mice, while FMT restores microbial balance and enhances gut motility

To ensure spatial precision in our TLI models, we employed micro-CT-guided field delineation with abdominal lead shielding to achieve precise lung targeting while minimizing off-target exposure (S4A and S4B Fig). Meanwhile, to definitively exclude confounding effects of abdominal radiation exposure, we conducted a comparative analysis of three irradiation protocols: partial apical lung irradiation (PALI) group, TLI group, and TLI without the abdominal lead shielding (TLAI) group. The result revealed mice in TLAI group developed acute gastrointestinal toxicity manifested as 83.3% mortality (S4C Fig), worsening diarrhea and weight loss (S4D Fig), intestinal hyperemia, and accelerated gut motility (S4E and S4F Fig). Intestinal AB-PAS histomorphology demonstrated severe mucosal barrier compromise with severe crypt-villus architecture disintegration in TLAI mice (S4G Fig). Conversely, PALI and TLI groups exhibited comparable gut motility and impaired mucosal barrier integrity without acute radiation-induced abdominal pathology. This directional divergence confirms that abdominal shielding in our TLI model effectively isolates direct abdominal radiation effects, while maximizing targeted induction of RILI.

**Fig 3:**
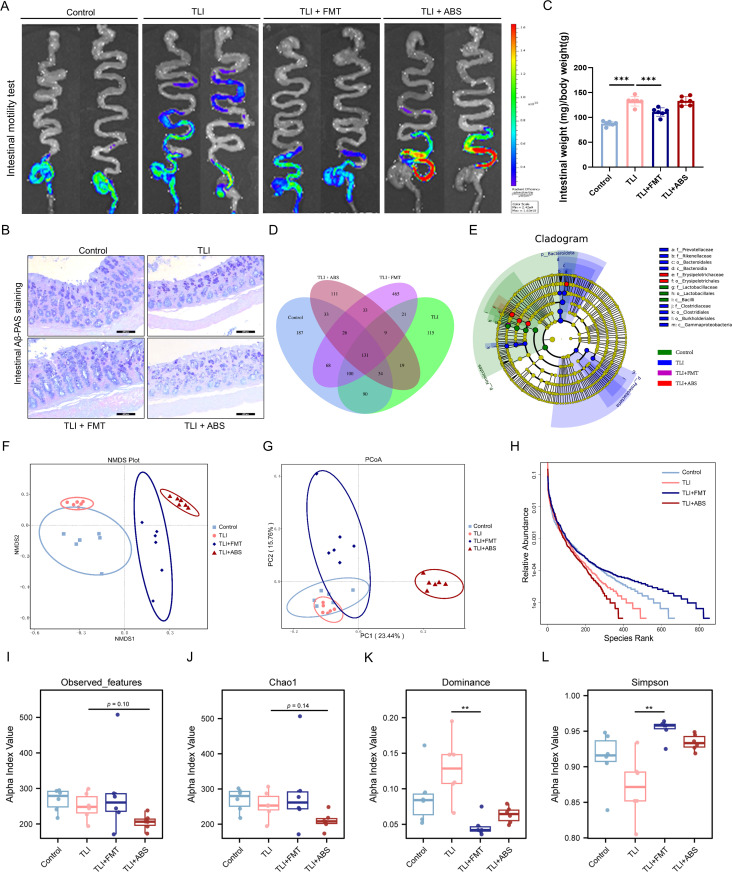
FMT restores gut microbiota composition and intestinal function in RILI mice. **(A)** Representative images of intestinal motility across groups. **(B)** Intestinal AB-PAS staining. **(C)** Ratio of intestinal weight to body weight. **(D)** Venn diagram of the distribution of shared and unique microbial taxa among four groups. **(E)** LEfSe cladogram of taxa. **(F)** The NMDS and (G) the PCoA plots across groups. **(H)** Rank-abundance curves across groups. **(I-L)** Alpha-diversity indices of Observed features, Chao1, Dominance and Simpson. n = 6 per group. Data are presented as mean ± SD (C) or median ± IQR **(I-L)**. Statistical comparisons were performed using one-way ANOVA with Tukey’s post hoc test **(C)**, or Kruskal-Wallis test with Dunn’s post hoc comparisons/Benjamini-Hochberg FDR adjustment **(I-L)**. **p < 0.01, ***p < 0.001.

Subsequently, we further investigate the impact of RILI on the gut microbiota, as well as on the structure and function of the gut, along with the gut motility test and mucosal barrier integrity in response to FMT and ABS intervention. The results revealed that TLI reduced gut motility and impaired mucosal barrier integrity, with concomitant elevation in intestinal relative weight (Fig 3A-3C). Interestingly, FMT partially ameliorated the decline in gut motility and function observed in TLI mice. 16S rRNA sequencing was performed to evaluate the impact of FMT on the gut microbiota of radiation-exposed mice to further elucidate the underlying mechanisms. The Venn diagram illustrates the shared and unique gut microbiota among the four groups of mice (Fig 3D). Additionally, the cladogram generated using LEfSe identified distinct taxonomic clusters among the four groups (Fig 3E). The NMDS and PCoA plots visualized the structural variance in gut microbiota among different groups (Fig 3F and 3G). These structural alterations were corroborated by complementary diversity metrics, including the Rank-abundance curve, Observed features, Chao1, Simpson and Dominance diversity indices. Critically, rank-abundance curves provided multidimensional insights into community structure, simultaneously capturing shifts in species richness and evenness. As evidenced in Fig 3H, the TLI + FMT group and Control group demonstrated a shallower curve slope relative to the TLI groups, indicating significantly higher species diversity and more equitable community structure. Conversely, the steepest slopes in TLI + ABS reflected marked reductions in diversity, further evidenced by its lowest Observed features (*p* = 0.10) and Chao1 indices (*p* = 0.14) (Fig 3I and 3J). Although statistically non-significant, their consistent direction and magnitude suggest biologically relevant trends. Additionally, TLI exhibited relatively elevated Dominance and reduced Simpson index versus Control, suggesting disrupted community structures in evenness and diversity (Fig 3K and 3L). Notably, FMT treatment reversed this TLI-induced dysbiosis, evidenced by restored Dominance and reduced Simpson indices (p < 0.01), indicating recovery of microbial homeostasis. However, the lack of significant change in the Simpson index for the ABS group may be attributed to compensatory dynamics of antibiotic-resistant taxa, which did not lead to a marked decrease. Complementing the diversity analyses, the microbial content assessment using a traditional nutrient agar plate, further confirmed FMT efficacy in mitigating radiation-induced gut microbiota dysbiosis (S5 Fig).

**Fig 4:**
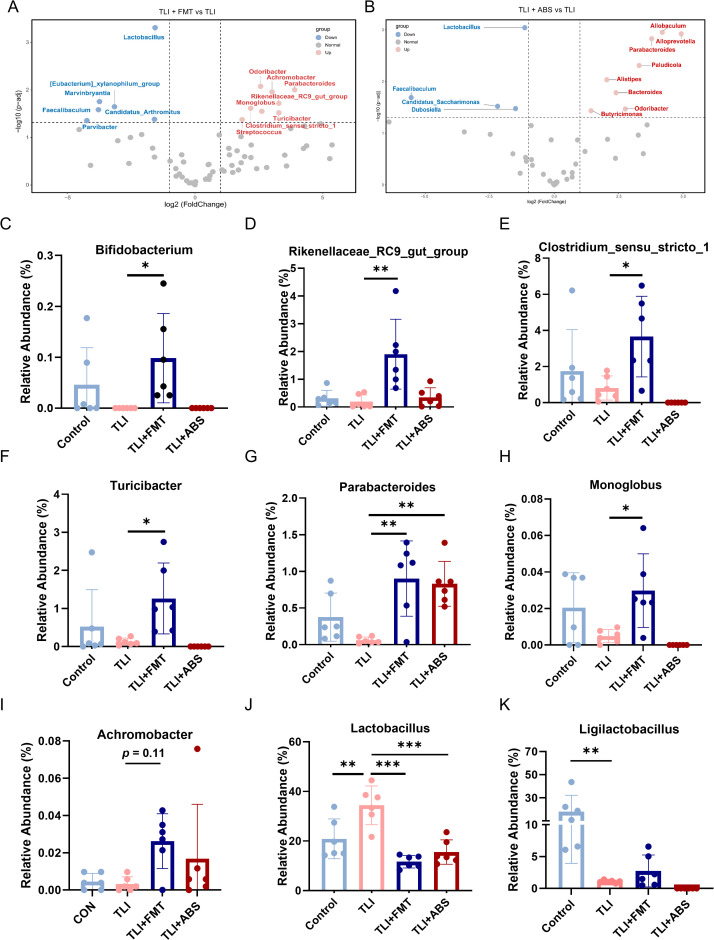
FMT alleviates gut microbiota dysbiosis induced by RILI. **(A-B)** Volcano plots visualizing differential taxa between indicated groups. **(C-K)** Relative abundance of specific bacterial taxa across groups, including *Bifidobacterium*, *Rikenellaceae RC9*, *Clostridium sensu stricto 1, Turicibacter*, *Parabacteroides, Monoglobus*, *Achromobacter*, *Lactobacillus* and *Ligilactobacillus*. n = 6 per group. Data are presented as mean ± SD. Statistical comparisons were performed using one-way ANOVA with Tukey’s post hoc test or Kruskal-Wallis test with Dunn’s post hoc test **(C-K)**. *p < 0.05, **p < 0.01, ***p < 0.001.

The volcano plot revealed significant gut microbiota compositional shifts between TLI + FMT and TLI groups, as well as between TLI + ABS and TLI groups, which may be closely associated with the TLI disease process (Fig 4A and 4B). However, zero-abundance bacteria cannot be visualized in these volcano plots. Bubble plots were utilized to provide complementary visualization of relative abundance differences across groups (S6 Fig). As showed in Fig 4C to 4K, FMT concurrently enriched numerous beneficial taxa including *Bifidobacterium*, *Rikenellaceae_RC9* and *Parabacteroides*, *Turicibacter* and so on. Among them, *Bifidobacterium* is well-known for their roles in enhancing gut barrier function and modulating the immune system, which can lead to improved gut motility and integrity [11]. Beyond these canonical gut-centric functions, *Bifidobacterium* has been reported to reduce hippocampal damage and improve memory in an ADHD rat model, suggesting a role in tissue repair [12]. *Rikenellaceae RC9*, a potential short-chain fatty acids (SCFAs)-producing bacterium, has been shown to alleviate inflammation in allergic asthma [13], reduce oxidative stress, and maintain gut homeostasis during cold acclimation [14]. Increased *Rikenellaceae* abundance has also been inversely correlated with pro-inflammatory cytokine production [15]. Additionally, *Parabacteroides* mediates multi-organ protection by suppressing inflammation and fibrosis via the modulation of host metabolic pathways [16,17]. Similarly, *Clostridium sensu stricto 1* is also positioned within the metabolic network, being functionally linked to the biosynthesis of amino acids and SCFAs, thereby contributing to immune regulation [18]. Additionally, while some studies link *Turicibacter* to the pathogenesis of inflammatory diseases [19,20], others suggest it may regulate gut microbiota composition and inhibit pathogenic bacteria growth [21]. Meanwhile, *Ligilactobacillus,* a reclassified genus from *Lactobacillus*, was significantly depleted in TLI mice versus controls. Existing evidence indicates *Ligilactobacillus* not only enhances intestinal metabolic functions and provides extraintestinal pathogen defense [22,23], but also mitigates distal tissue inflammation and oxidative damage through gut-immune axis coordination [24–26]. Intriguingly, our study found that *Lactobacillus* abundance increased significantly in RILI mice, a phenomenon partially reversed by FMT. This disease-associated enrichment pattern of *Lactobacillus* aligns with clinical and experimental observations in inflammatory conditions including rheumatoid arthritis [27], collagen-induced arthritis [28], and inflammatory bowel disease [29,30]. Collectively, these microbiome changes suggest that FMT may restore gut ecological equilibrium, and shift the dysbiotic community toward a homeostatic state.

### FMT mitigates radiation-induced gut fatty acid metabolism disruption in RILI mice

**Fig 5:**
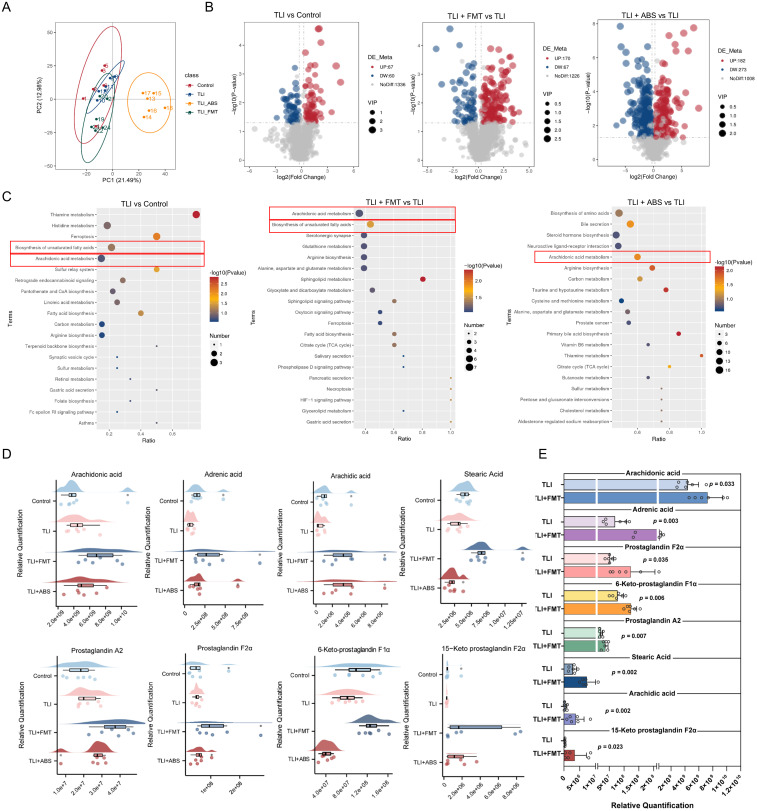
FMT restores unsaturated fatty acid synthesis and arachidonic acid metabolism in RILI mice. **(A)** Principal component analysis (PCA) of distinct metabolic profiles among groups. **(B)** Volcano plots showing differentially expressed metabolites between indicated comparison groups. **(C)** Metabolite enrichment analysis identifying significantly affected pathways in indicated comparison groups. **(D)** Rain cloud plot showing the metabolic profiles of unsaturated fatty acids and arachidonic acid pathway metabolites across group, including arachidonic acid, adrenic acid, arachidic acid, stearic acid, prostaglandin A2, Prostaglandin F2α, 6-Keto-prostaglandin F1α, 15-Keto prostaglandin F2α. **(E)** Relative quantification of the metabolites in TLI + FMT vs TLI groups. n = 6 per group. Data are presented as mean ± SD. Statistical comparisons between indicated groups were performed using two-sided unpaired Student’s t-tests **(E)**. * *p* < 0.05, ** *p* < 0.01, *** *p* < 0.001.

To characterize gut metabolic profiles under normal and RILI conditions, as well as after FMT and ABS interventions, we performed untargeted liquid chromatography-mass spectrometry (LC-MS) to identify and quantify fecal metabolites. Metabolomics analysis revealed that RILI induces significant disruptions in gut fatty acid metabolism in mice, particularly reducing unsaturated fatty acid synthesis and disrupting arachidonic acid metabolism. Principal component analysis (PCA) showed significant differences in gut metabolic profiles among the four groups of mice (Fig 5A). Compared to the control, TLI induced the upregulation of 67 metabolites and the downregulation of 60 metabolites. Following FMT treatment in comparison to TLI, there was a significant elevation in 170 metabolites and a decrease in 67. Additionally, the ABS group exhibited more extensive changes relative to TLI, with 182 metabolites upregulated and 273 downregulated (Fig 5B). The results demonstrate notable shifts in the gut metabolic landscape of RILI mice and underscore the significant impact of gut microbiota interventions on these radiation-induced metabolic alterations. MetKEGG enrichment analysis indicated that differential metabolites in the TLI group were closely linked to unsaturated fatty acids and arachidonic acid metabolism. Similarly, significant enrichment in these metabolic pathways was also observed in the FMT treatment group. Notably, the differential metabolites of TLI + ABS group further highlighted the compromised homeostasis in the arachidonic acid metabolism pathway (Fig 5C). We further employed MSEA analysis to determine the functional state (S7 Fig). The results showed that the arachidonic acid metabolism pathway was negatively enriched in the TLI group compared to controls, indicating suppression following radiation-induced injury (TLI vs Control, NES = -1.75). FMT treatment reversed this suppression, resulting in positive enrichment (TLI + FMT vs TLI, NES = 1.05). In contrast, ABS intervention did not restore this pathway functionality, which remained in a negatively enriched state (TLI + ABS vs TLI, NES = -1.25). Specifically, the metabolic pathways enriched by FMT treatment, mainly included metabolites such as arachidonic acid, adrenic acid, arachidic acid, stearic acid, prostaglandin A2, prostaglandin F2, 6-keto prostaglandin F1α and 15-keto prostaglandin F2α (Fig 5D and 5E). These findings clearly demonstrate that FMT confers protection by remodeling the gut microbiota and positively activating metabolic pathway, transforming it from an injury-associated suppressed state into a functionally active and protective state. Conversely, ABS intervention, although capable of perturbing metabolism, failed to induce beneficial functional metabolic reprogramming.

As previously mentioned, FMT treatment led to a significant increase in the relative abundance of beneficial bacterial taxa. These probiotics promote the synthesis and maintenance of unsaturated fatty acids by facilitating the conversion of dietary fatty acids or modulating host metabolic pathways. *Rikenellaceae RC9*, a potential SCFAs-producing bacterium, has been found to alleviate inflammation in allergic asthma [13], reduce oxidative stress, and maintain gut homeostasis during cold acclimation [14]. Increased *Rikenellaceae* abundance has also been inversely correlated with pro-inflammatory cytokine production [15]. *Clostridium sensu stricto 1* produces SCFAs that positively impact gut health [18]. *Bifidobacterium* has been reported to reduce hippocampal damage and improve memory in an ADHD rat model, suggesting a role in tissue repair, although this effect remains debated [12]. Additionally, while some studies link *Turicibacter* to the pathogenesis of inflammatory diseases [19,20], others suggest it may regulate gut microbiota composition and inhibit pathogenic bacteria growth [21].

### FMT promotes ER-phagy in radiation-induced lung injury mice

**Fig 6:**
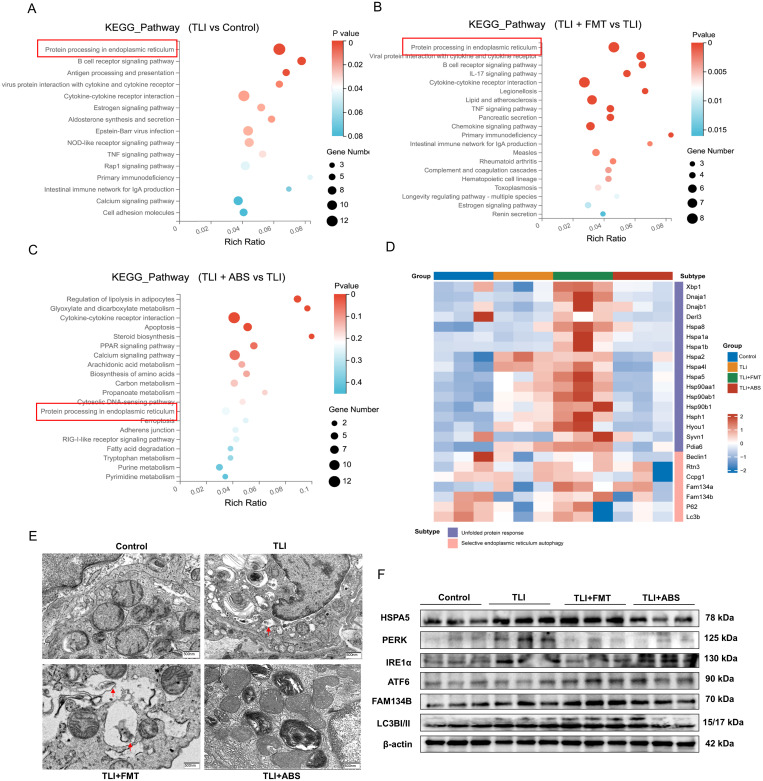
Radiation-induced disruption of ER protein processing in lung tissue and enhanced ER adaptive response by FMT (A-C) KEGG pathway enrichment analysis showing significant enrichment of protein processing in endoplasmic reticulum in differentially expressed genes across indicated comparison groups. **(D)** Heatmap illustrating the differential expression of key ER protein processing-related genes associated with the adaptive ER response across groups. **(E)** Transmission electron microscopy images showing ER morphology across groups. **(F)** Western blot of ER adaptive response-related proteins, including HSPA5, PERK, IRE1α, ATF6, FAM134B, and LC3B I/II, using lung tissue from mice. n = 3 per group.

The endoplasmic reticulum (ER) is crucial for protein synthesis, folding, and quality control, serving as a key component in the maintenance of cellular homeostasis. It is also involved in regulating various processes, including inflammation, autophagy, and apoptosis [31]. ER-phagy supports homeostasis by degrading damaged ER fragments and misfolded proteins [32]. When irradiation-induced DNA damage and oxidative stress impair the ER’s protein folding capacity, unfolded proteins accumulate. This triggers self-rescue mechanisms that restore ER homeostasis and reduce inflammation. Transcriptomic analysis revealed significant enrichment of the protein processing pathway in the ER across Control vs. TLI (*p* = 0.001) and FMT vs. TLI (*p* = 2e-5) comparisons (Fig 6A and 6B), while no significant enrichment was observed in TLI + ABS vs. TLI (*p* = 0.237, Fig 6C). The heatmap results show that FMT treatment further increased gene expression within this pathway, while ABS treatment suppressed its expression levels (Fig 6D). Transmission electron microscopy (TEM) revealed that radiation-induced pronounced expansion and swelling of ER structures in lung tissues, while FMT treatment led to partial restoration of ER morphology, with selective autophagosome encapsulation of certain ER regions containing dense punctate or granular aggregates, indicative of ongoing ER degradation. In contrast, the ABS group also exhibited noticeable ER expansion and vesiculation, with no apparent signs of selective autophagosome encapsulation (Fig 6E).

In summary, FMT effectively restored ER function and promoted ER-phagy in response to radiation-induced ER stress, further enhancing UPR-related markers and suggesting a protective role in maintaining cellular homeostasis under stress conditions. These findings provide crucial experimental evidence for understanding the mechanisms underlying radiation damage prevention and treatment, highlighting the therapeutic potential of FMT in mitigating RILI.

### Multi-omics analysis reveals arachidonic acid metabolism may improve RILI by promoting ER-phagy

**Fig 7:**
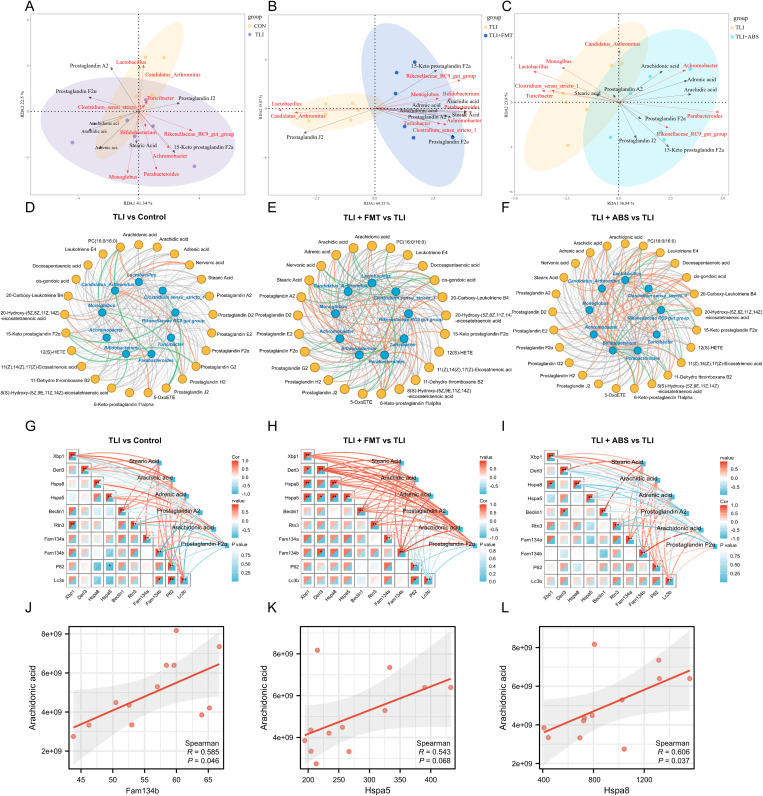
Multi-omics analysis of gut microbiota, metabolite, and lung transcriptome interactions. **(A-C)** Contribution of gut microbiota to the levels of metabolites with microbiota represented by red arrows and metabolites by black arrows. Acute inter-arrow angles indicate positive correlations. n = 6 per group. **(D-E)** Key bacterial genus and metabolite correlations in the gut-lung axis. Orange lines: positive correlations. Green lines: negative correlations. Gray lines: non-significant correlations. n = 6 per group. **(G-I)** Correlation network heatmaps of unsaturated fatty acid metabolites and ER-related genes across groups in different comparisons. n = 3 per group. **(J–L)** Scatter plots showing correlations between arachidonic acid levels and ER-related genes (Fam134b, Hspa5 and Hspa8) in mice with matched transcriptomic-metabolomic profiles. n = 3 per group.

To characterize the interplay between gut microbiota, metabolites, and gene expression levels, we analyzed the correlations among these parameters via using multi-omics analysis. RDA analysis explored the potential contribution of microbiota to metabolite distribution, and the results indicate that variations in metabolite distribution are largely driven by the microbiota (Fig 7A-7C). By comparing the distribution across different groups, our results further highlight the correlation between the microbiota and key metabolites (Fig 7D-7F). Notably, *Bifidobacterium*, *Parabacteroides, Achromobacter*, *Clostridium_sensu_stricto_1* and *Rikenellaceae_RC9 _gut_group*, showed significant associations with a range of arachidonic acid (AA) and downstream metabolites. In addition, the correlation network heatmap reveals relationships among ER adaptive response-related genes across paired comparisons and their associations with key metabolites involved in unsaturated fatty acid and AA metabolism (Fig 7G-7I). The network heatmap highlights the interactions among genes involved in ER protein processing and illustrates the relationships between these genes and metabolite nodes. The color of the network lines represents the correlation coefficient (r value) between genes and metabolite nodes, while the grid color in the heatmap indicates the correlation coefficient (Cor) between genes. In the comparison between the FMT group and the TLI group, Hspa8 showed significant positive correlations with both Xbp1 and Hspa5 (Cor = 0.943, *p* = 0.0048), and Derl3 was significantly positively correlated with Fam134b (Cor = 0.89, *p* = 0.0188). P62 and Lc3b exhibited a significant positive correlation (Cor = 0.89, *p* = 0.0188), suggesting their potential synergistic role in ER adaptive responses. Correlation analysis between metabolites and genes, visualized in scatter plots, indicated varying degrees of positive correlation between AA and ER adaptive response-related genes across the four groups. Specifically, AA showed a significant positive correlation with Fam134b (r = 0.585, *p* = 0.046) and with Hspa8 (r = 0.606, *p* = 0.037). While correlations between Hspa5 (r = 0.543, *p* = 0.068) and Xbp1 (r = 0.529, *p* = 0.077) with arachidonic acid did not reach statistical significance, they nonetheless indicated a strong positive trend (Fig 7J-7L). Overall, the significant positive correlations between arachidonic acid and these ER-phagy-related genes further support its potential regulatory role in RILI and FMT treatment, suggesting a critical function for arachidonic acid in ER stress and autophagy processes during RILI.

### AA enhances DNA damage repair and ER-phagy in irradiated lung epithelial cells

**Fig 8:**
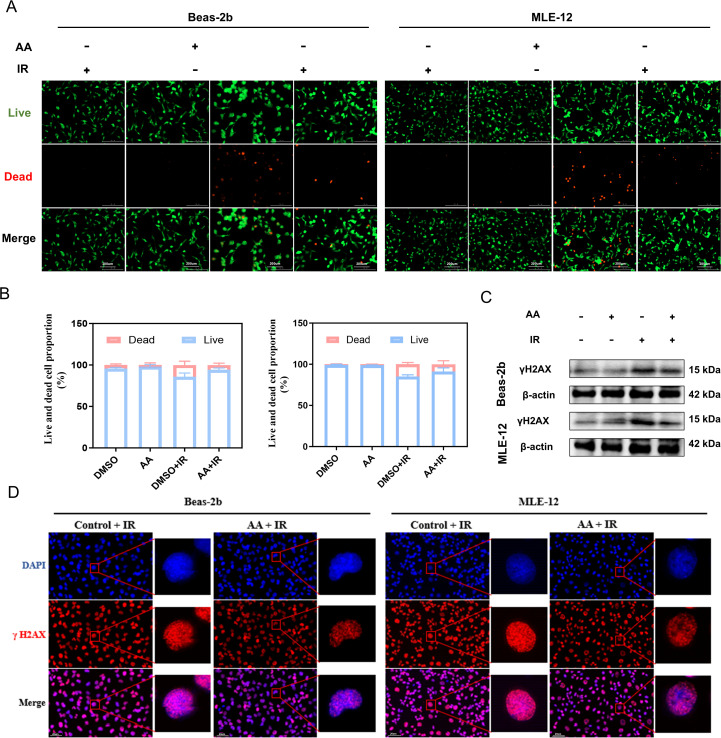
Arachidonic acid enhances DNA repair in irradiated lung epithelial cells. **(A)** Cell viability and death assays indicating that AA treatment reduces radiation-induced cell death. **(B)** Proportions of live (blue) and dead (red) cells across treatment. **(C)** Western blot showing decreased γH2AX expression of DNA repair proteins in irradiated lung epithelial cells treated with AA. **(D)** γH2AX foci analysis showing reduced DNA double-strand breaks in AA-treated irradiated cells. n = 6 per group.

Building on the findings from the multi-omics analysis, we investigated the differences in serum AA levels among various groups of mice. The results demonstrated that FMT can simultaneously elevate serum concentrations in mice with RILI (S8 Fig). To dissect the underlying cellular mechanisms, we employed an *in vitro* model of irradiated lung epithelial cells. The optimal AA concentration for subsequent experiments was determined using a CCK-8 dose-response assay (S9 Fig). We then evaluated the cytoprotective effects of AA on cell survival and DNA damage repair. Live-dead staining revealed that AA significantly enhanced the survival of irradiated cells relative to untreated controls (Fig 8A and 8B). Western blot analysis confirmed decreased γH2AX expression in AA-treated cells (Fig 8C). We further examined the repair of DNA double-strand breaks by monitoring γH2AX foci at 3 and 12hours post-irradiation. AA-treated cells exhibited a pronounced reduction in foci as early as 3 hours (Fig 8D), with further decline by 12 hours (S10 Fig), collectively indicating that AA accelerates the repair of radiation-induced DNA damage.

**Fig 9:**
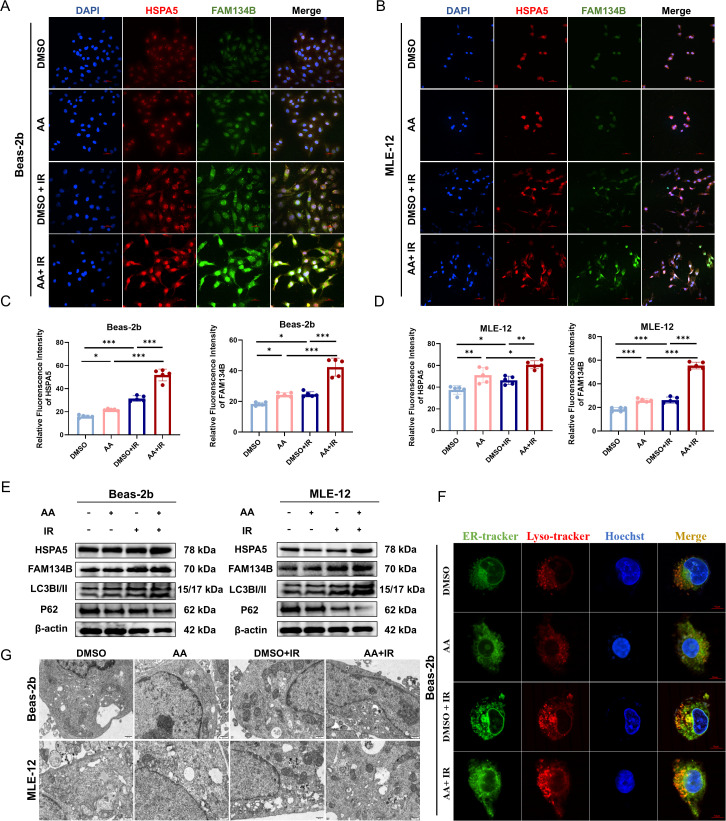
Effects of arachidonic acid on ER-phagy in irradiated lung epithelial cells. **(A-B)** Immunofluorescence of HSPA5 (red) and FAM134B (green) in Beas-2b and MLE-12 cells. **(C-D)** Quantification of fluorescence intensity for HSPA5 and FAM134B in Beas-2b and MLE-12 cell lines. n = 5 per group. **(E)** Western blot of HSPA5 and FAM134B expression across groups in Beas-2b and MLE-12 cell lines. n = 6 per group. **(F)** Representative confocal microscopy images of ER (green) and lysosome (red) distribution across experimental groups. n = 6 per group. **(G)** Transmission electron microscopy images depicting ER morphology across groups. n = 6 per group. Data are presented as mean ± SD. Statistical comparisons were performed by one-way ANOVA with Tukey’s post hoc test **(C-D)**. * *p* < 0.05, ** *p* < 0.01, *** *p* < 0.001.

Given the *in vivo* evidence of ER-phagy activation and the multi-omics data suggesting a correlation between AA and adaptive ER responses, we further investigated this pathway *in vitro*. Immunofluorescence analysis demonstrated that AA treatment upregulated adaptive ER chaperone protein HSPA5 and the selective autophagy receptor FAM134B in irradiated cells (Fig 9A-9D). Western blot analysis confirmed the increased expression of HSPA5 and FAM134B, indicating that AA facilitates the ER adaptive responses and selective autophagy, effectively mitigating radiation-induced cell damage (Fig 9E). To further substantiate these observations, ER-tracker and Lyso-tracker staining assays demonstrated that AA treatment significantly increased the extent of ER-phagy in irradiated lung epithelial cells (Fig 9F). Transmission electron microscopy indicated that AA alleviates radiation-induced ER expansion and promotes the formation of ER phagosome (Fig 9G). In conclusion, our findings demonstrate that AA drives protective ER-phagy in irradiated lung epithelial cells, supporting an adaptive response to radiation and facilitating the repair of radiation-induced cellular damage.

### AA transcriptionally upregulates FAM134B via PPARγ to induce ER-phagy

**Fig 10:**
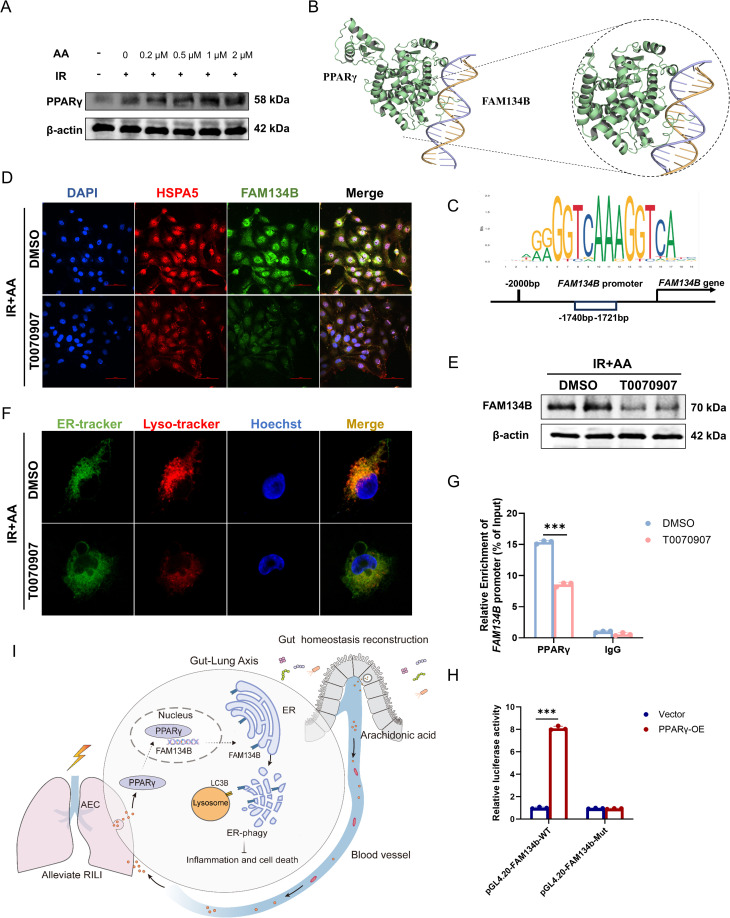
Arachidonic acid upregulates FAM134B-mediated ER-phagy in a PPAR γ-depended manner. **(A)** Western blot of PPARγ expression in varying concentration of AA under irradiation. **(B)** Molecular docking analysis between PPARγ and the fam134b promoter. **(C)** Immunofluorescence staining of HSPA5 (red) and FAM134B (green) in Beas-2b cells with or without following PPARγ inhibitor (T0070907) administration. **(D)** Representative confocal microscopy images of ER (green) and lysosome (red) distribution across groups. **(E)** Western blot of FAM134B expression between the T0070907-treated group and the control group. **(F)** Consensus DNA binding motif for PPARγ. **(G)** ChIP-qPCR analysis of PPARγ occupancy at the Fam134b promoter region in cells treated with 10μM T0070907 or DMSO control. **(H)** Dual-luciferase reporter assay of PPARγ transactivation at the Fam134b promoter. **(I)** Schematic mechanism of gut microbiota-derived arachidonic acid mediates PPARγ-dependent activation of FAM134B-mediated ER-phagy to ameliorate RILI. n = 3 per group. Data are presented as mean ± SD. Statistical comparisons were performed by two-way ANOVA with Tukey’s post hoc test **(G-H)**. * *p* < 0.05, ** *p* < 0.01, *** *p* < 0.001.

We then sought to delineate the mechanism by which AA upregulates FAM134B. PPARγ, a nuclear receptor tightly associated with oxidative stress, has been intensively investigated in strategies aimed at protecting against lung injury [33,34]. We observed that PPARγ increased in expression in a dose-dependent manner with AA treatment and was further enhanced by IR exposure (Fig 10A). As shown in S11 Fig, molecular docking simulations of AA with PPARγ suggested a high likelihood of direct binding (binding affinity = -7.1 kcal/mol). Given that PPARγ is nuclear-localized and can be activated via direct binding to AA, we used the JASPAR database and molecular docking simulations to predict that PPARγ as a potential transcriptional regulator of FAM134B with stable interaction at the Fam134b gene promoter (Fig 10B and 10C). Arachidonic acid-treated irradiated pulmonary epithelial cells were also treated with the PPARγ inhibitor T0070907. Immunofluorescence and Western blot analysis confirmed a significant reduction in FAM134B expression in the T0070907-treated group (Fig 10D and 10E). Additionally, ER-Tracker and Lyso-Tracker staining revealed a decrease in ER-phagy in irradiated lung epithelial cells treated with the PPARγ inhibitor (Fig 10F). Finally, based on the consensus DNA binding motif for PPARγ, ChIP assays and dual-luciferase reporter assay confirmed that PPARγ directly binds to the FAM134B promoter, establishing it as a transcriptional activator of FAM134B (Fig 10G and 10H).

Collectively, this study demonstrates that FMT reshapes gut homeostasis in RILI mice, thereby enhancing AA metabolism to activate the nuclear receptor PPARγ, which directly binds the FAM134B promoter, transcriptionally upregulating FAM134B expression to induce ER-phagy, ultimately alleviating RILI by facilitating the clearance of damaged endoplasmic reticulum (Fig 10I).

### AA activates protective ER-phagy and attentuates RILI in mice

To investigate the impact of AA on RILI and to identify its optimal therapeutic dose, we conducted a dose-ranging study in a murine model. Wild-type C57BL/6 mice were subjected to thoracic irradiation to establish the RILI model. 24 hours post-irradiation, the mice were orally administered AA at doses of 10, 50, 100, or 200 mg/kg once daily for 14 days. The severity of lung injury, assessed by H&E and Masson staining, revealed that AA at 100 mg/kg provided the greatest protective efficacy against RILI (S12A Fig). We further measured oxidative stress markers, including GSH and MDA, alongside pulmonary edema and body weight changes in mice (S12B-S12E Fig). The findings likewise supported that an in vivo intervention dose of 100 mg/kg AA confers a marked improvement in TLI mice, providing a basis for dose selection in subsequent experiments.

**Fig 11:**
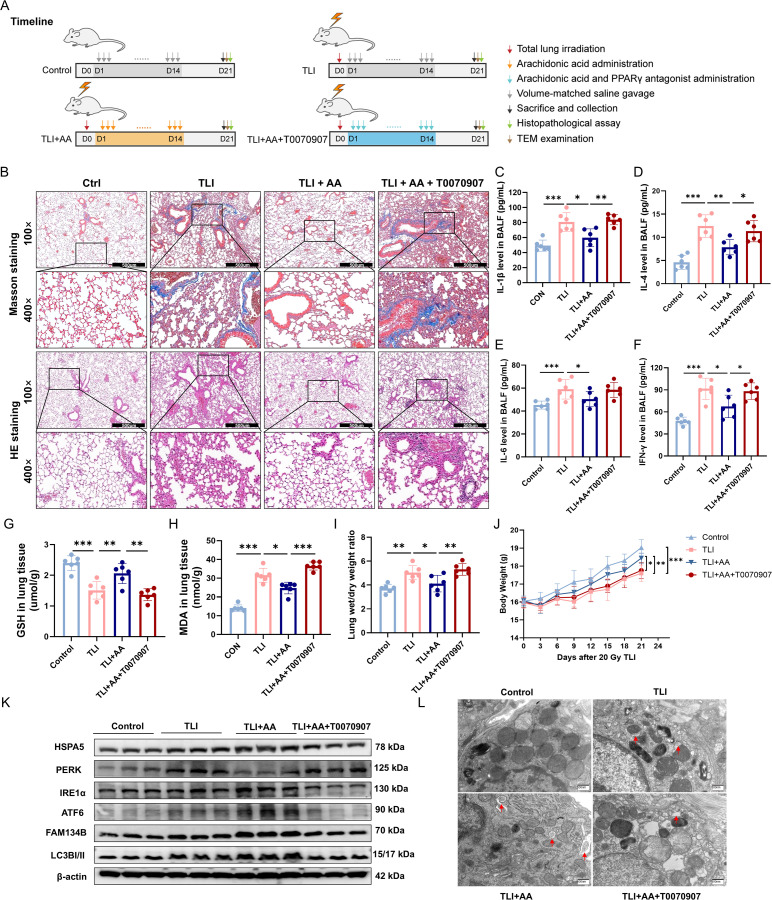
Arachidonic acid protects against radiation induced lung injury in mice by Activating PPAR γ. **(A)** The timeline of experimental interventions and sampling. **(B)** Representative Masson’s trichrome, and H&E staining of lung tissues (100× and 400×). **(C-F)** BALF levels of IL-1β, IL-4, IL-6 and IFN-γ quantified by ELISA. **(G, H)** GSH and MDA levels of lung tissue. **(I)** Lung wet/dry weight ratio across groups. **(J)** The change of body weight of each experimental mice after 20 Gy TLI. n = 6 per group. **(K)** Western blot of ER adaptive response-related proteins, including HSPA5, PERK, IRE1α, ATF6, FAM134B, and LC3B I/II, using lung tissue from mice. n = 3 per group. **(L)** Transmission electron microscopy images showing ER morphology across groups. Data are presented as mean ± SD. Statistical comparisons were performed using one-way ANOVA with Tukey’s post hoc test. * p < 0.05, ** p < 0.01, *** p < 0.001.

Having established 100 mg/kg as the optimal dose, we next comprehensively evaluated the therapeutic effect of AA on RILI, incorporating the PPARγ inhibitor T0070907 as a regulatory condition to verify the target role of PPARγ (Fig 11A). We found that AA treatment significantly alleviated radiation-induced lung histopathological damage (Fig 11B), reduced inflammatory cytokines IL-1β, IL-4, IL-6, IFN-γ in BALF (Fig 11C-11F), mitigated oxidative stress (Fig 11G and 11H), and improved pulmonary edema and overall health status (Fig 11I and 11J). Critically, at the molecular level, AA treatment in mice upregulated key proteins governing the ER adaptive response as represented by HSPA5 and ATF6, as well as mediators of ER-phagy FAM134B and LC3B-II, as evidenced by Western blot (Fig 11K). Most importantly, examination of lung tissues by TEM revealed that AA alleviated radiation-induced ER dilation and promoted the formation of protective ER-phagosomes (Fig 11L). These *in vivo* results establish that AA activates a protective ER-phagy program in RILI, facilitating tissue repair. Co-administration of a PPARγ antagonist fully reversed these protective effects, indicating a PPARγ-dependent mechanism consistent with our in vitro findings, in which AA activated PPARγ to induce FAM134B-mediated ER-phagy in lung epithelial cells.

## Discussion

This study underscores the bidirectional communication of the gut-lung axis as a critical regulator in RILI, highlighting the therapeutic potential of targeting gut microbiota-derived metabolites for RILI protection. In our RILI mice model, despite abdominal shielding during irradiation, we observed disrupted intestinal functional homeostasis (reduced intestinal motility, impaired barrier integrity, and metabolic dysregulation) and microbial community structure through systemic inflammation and oxidative stress. However, FMT effectively mitigated subsquent damage and promoted pulmonary repair by remodeling gut microbiota and metabolic balance. These findings position gut microbiota dysbiosis as an exacerbating factor or disease contributor in RILI pathogenesis. Although was not confirmed as the direct damage factor, subsequent gut dysbiosis exacerbates tissue injury through disruption of gut-lung axis signaling. This bidirectional crosstalk likely involves circulating bioactive molecules, thereby linking gut-lung interactions with tissue homeostasis and inflammatory regulation.

FMT has emerged as a core therapeutic strategy for multiple dysbiosis-related diseases through restructuring the gut microbiota, including infections, gut diseases, gut-organ axis disorders and other diseases [35]. In this study, we investigated the therapeutic potential of FMT in RILI mice model to elucidate its impact through the gut-lung axis. 16S rRNA sequencing revealed altered microbial structure in RILI mice compared to controls, with reduced rank abundance indicating a disrupted community structure characterized by decreased evenness and diversity. Importantly, FMT intervention reversed the overall dysbiosis induced by TLI, as evidenced by restored Dominance and Simpson indices, suggesting a recovery of microbial balance. Specifically, FMT enriched multiple beneficial taxa, such as *Bifidobacterium*, *Rikenellaceae_RC9* and *Parabacteroides*, *Turicibacter*, and others. These beneficial taxa are implicated in enhancing gut barrier function, modulating the immune system, and promoting metabolic homeostasis [12–16]. Additionally, FMT reversed the TLI-induced expansion of *Lactobacillus*, a finding that aligns with the characteristic *Lactobacillus* overgrowth seen in several inflammatory diseases [28–30]. Concurrently, improved gut motility and barrier integrity were also observed in RILI mice following FMT treatment. The restoration of microbial structural homeostasis, coupled with improved intestinal functionality, provides enhanced evidence for the functional coordination operating between gut microbiota and the host.

Microbial metabolites have been established as critical mediators in host-microbiota crosstalk [36]. Previous evidence indicates that these metabolites can mediate abscopal effects via immune and humoral routes to influence the progression and repair of radiation-induced tissue injury [37,38]. Our metabolomic analysis revealed that RILI induced significant metabolic dysregulation. The restoration of gut microbial ecology following FMT was closely linked to the recovery of host metabolic homeostasis, particularly reflected in pathways of unsaturated fatty acid and AA metabolism. Given the central role of AA as an essential omega-6 polyunsaturated fatty acid at the convergence of these pathways, we therefore focused on it as a potential mediator of the observed protection. This proposition is supported by prior evidence indicating that AA and its downstream metabolites facilitate tissue repair across multiple organs [39–41]. Furthermore, our subsequent *in vivo* experiments confirmed that AA supplementation effectively ameliorates RILI in a concentration-dependent manner. These functional validation results corroborate our metabolomics findings. Although AA may not be the exclusive mediator of FMT-conferred protection, it is unequivocally identified as one of the most critical components, based on its significant differential abundance and established biofunctions. Therefore, the restoration of microbial balance by FMT in RILI initiates a restorative cascade, from the recovery of intestinal function to metabolic homeostasis, which establishes a basis for its distal protective potential.

Integrated multi-omics analysis and ultrastructural observations reveal that RILI coincided with significant disruption of ER protein processing pathways in lung tissue, accompanied by marked ER dilation and vacuolization in pulmonary epithelial cells following irradiation. Notably, FMT activated these pathways and enhanced ER adaptive responses. Further morphological and biomarker analyses demonstrated that both FMT and AA administration reversed these disturbances by effectively activating ER-phagy, a protective autophagic process that clears damaged ER fragments via lysosomal degradation to reestablish proteostasis. As the central hub for protein synthesis, folding and quality control, the ER is a pivotal regulator of inflammation, autophagy and apoptosis [31]. Disruption of ER homeostasis is a well-recognized mechanism in acute solid organ injury [42,43], and the activation of ER-phagy represents an important strategy for achieving early organ repair [44,45]. Mechanistically, RILI-induced systemic inflammation and oxidative stress perturb gut ecological integrity and diversity, thereby disrupting an unsaturated fatty acid metabolic network centered on AA. By remodeling the gut microbiota and associated lipid metabolic networks, FMT promotes ER adaptive responses and activates selective autophagy in alveolar epitheliums, leading to coordinated mitigation of RILI at both systemic and organelle levels. Collectively, these findings support the existence of a coherent “gut microbiota-lipid metabolism-ER adaptation” axis that underlies the observed phenotypic improvement.

Building on these findings regarding differential expression of ER adaptive response genes, elucidating the transcriptional programming at the level of nuclear receptors would establish a molecular basis for precise microbiota-metabolite interventions. PPARγ, as one of the main receptors for AA, plays a broad role in regulating lipid metabolism [46], inflammation [47], cell repair [48], and stress responses [49]. Within a nuclear receptor-centric framework governed by PPARγ, our findings demonstrate that AA functions as a bioactive lipid ligand to activate PPARγ, integrating lipid metabolism, immune homeostasis, and organelle quality control to drive adaptive responses during RILI. FAM134B, an essential ER‑phagy receptor, recruits autophagic machinery to degrade damaged ER regions and maintain ER quality control [50]. Mechanistically, AA-PPARγ signaling directly occupies the FAM134B promoter and enhances its transcription, augmenting selective clearance of injured ER fragments and establishing PPARγ as a transcriptional activator of FAM134B. In parallel, HSPA5, a core chaperone of the ER-adaptation response, mitigates protein‑folding stress and cooperates with FAM134B in damaged ER recognition and autophagic cargo assembly, forming a key execution node for ER‑phagy [51]. In our models, both AA supplementation and FMT increased HSPA5 and FAM134B expression, heightened ER‑phagy activity, alleviated oxidative stress, and enhanced DNA repair capacity, implicating the PPARγ-FAM134B axis as pivotal for re‑establishing organelle homeostasis. Consistent with multi‑omics associations, AA levels positively correlated with Fam134b and Hspa5 in lung tissue, supporting a transcriptionally programmed hub coupling microbiota, lipid metabolism, and ER adaptation. Given PPARγ’s broad roles in lipid metabolism, inflammation resolution, and energy homeostasis, targeting AA-PPARγ axis offers a strategy for protective ER‑phagy to mitigate RILI and foster tissue repair.

However, this study also has its limitations. The mouse model of whole-lung irradiation used in this study may not fully replicate the complexity of human RILI, particularly with regard to the long-term progression of the disease and the variability in patient responses. Furthermore, while this study has identified AA as a key mediator in FMT-mediated ER homeostasis in lung tissue, we cannot exclude the possibility that other metabolites contribute to complementary protective mechanisms. Nevertheless, our findings suggest that gut microbiota-derived metabolite modulation holds promise as a therapeutic strategy for radiation-induced injury and other disorders exhibiting impaired ER homeostasis.

In summary, the study establishes the gut-lung axis as a pivotal regulator of RILI and identifies FMT as an effective intervention that can act through metabolic remodeling and organelle-level repair mechanisms. The key outcome of this metabolic remodeling is the activation of the nuclear receptor PPARγ, leading to the up-regulation of the ER-phagy receptor FAM134B. This process restores ER homeostasis and enhances the repair capacity of lung epithelial cells, ultimately alleviating RILI. These findings delineate an integrated gut microbiota-metabolite-organelle network, providing a mechanistic foundation for targeting the gut-lung axis in RILI treatment.

## Materials and methods

### Ethics approval and consent to participate

The procedures of this study were approved and consented by the Animal Experimental Ethical Inspection of School of Public Health of Jilin University (No. SY202310002).

### Animals

All protocols were approved by the Animal Experimental Ethical Inspection of School of Public Health of Jilin University, and all animals were handled in accordance with its guidelines. C57BL/6J mice, aged 6–8 weeks (approximately 18 g), were obtained from Beijing Huafukang Biotechnology Co., Ltd. (Beijing, China). Mice were maintained in standard specific pathogen-free conditions (22 ± 2°C, 40–70% humidity, and a 12-hour light/dark cycle) with free access to standard chow and water. After an acclimatization period of 7 days, mice were randomly allocated into different experimental groups.

### Animal model and grouping

The schematic and timeline of the animal experiment design are presented in Figs 1 and 2A, respectively. To clarify the role and mechanism of FMT in alleviating early RILI, the experimental groups comprised the following four groups: Control, TLI, TLI + FMT and TLI + ABS. Mice in all groups except the Control group received standardized 20 Gy thoracic irradiation targeting consistent anatomical sites. To deplete gut microbiota prior to intervention, TLI + FMT group recipient mice were administered an antibiotic cocktail (1 g/L ampicillin, 0.5 g/L neomycin, 0.5 g/L vancomycin, 1 g/L metronidazole) in drinking water for five consecutive days, with a mean daily intake of 5–7 mL per mouse [52]. Twenty-four hours prior to performing FMT, the antibiotic cocktail was replaced with sterile water to eliminate residual effects. Subsequently, mice received oral gavage of 200 μL of a microbiota suspension in reduced physiological saline (saline supplemented with 0.5 g·L ⁻ ¹ L-cysteine and 0.2 g·L ⁻ ¹ Na₂S) once daily for 14 consecutive days after irradiation. The TLI + ABS group was treated with non-absorbable antibiotics (450 mg/L neomycin and 150 mg/L polymyxin B) in drinking water initiated 24 hours post-TLI and maintained for 14 days [53,54]. Mice in the Control and TLI groups received oral gavage of reduced physiological saline at an equivalent volume for 14 days. Mice were sacrificed and tissues collected after 3 weeks of lung irradiation. Meanwhile, we supplemented the experiment observation for 8 weeks after the irradiation. The irradiation and treatment methods were the same as before.

For murine models with varying pulmonary irradiation fields, experimental groups were established as follows: (1) PALI group received 20 Gy irradiation of the apical lung region (approximately 30% of total lung volume by pre-irradiation surface mapping), with complete abdominal shielding. (2) TLI group equal dose of whole-lung irradiation, with complete abdominal shielding. (3) TLAI group received 20 Gy irradiation exposure of both the upper abdomen and entire lung with shielding other non-target regions, administered at the same prescribed dose.

To evaluate the necessity of antibiotic preconditioning for FMT, we established four experimental groups: TLI, TLI + pre-Abx, TLI + FMT, and TLI + FMT/no pre-Abx. All groups received 20 Gy thoracic irradiation. The TLI + pre-Abx group were administered a 5-day broad-spectrum antibiotic cocktail preconditioning regimen prior to irradiation. The TLI + FMT group received the same 5-day antibiotic preconditioning, followed by FMT via oral gavage once daily for 14 consecutive days, starting 24 hours post-irradiation. The TLI + FMT/no pre-Abx group received FMT on the same schedule but without any prior antibiotic pretreatment. Tissues were collected 21 days after irradiation for subsequent analysis.

To identify the optimal therapeutic dose of AA, we conducted a dose-ranging study in a murine model of radiation-induced lung injury. Wild-type C57BL/6 mice receiving thoracic irradiation were randomly divided into five groups (n = 6): TLI and TLI + AA at 10, 50, 100, or 200 mg/kg. AA was dissolved in 8% *v/v* DMSO saline solution and administered by oral gavage once daily for 14 consecutive days, commencing 24 hours post-irradiation. Mice in the TLI group received an equivalent volume of the vehicle (8% *v/v* DMSO saline solution) on the same schedule. Based on optimal protection observed at 100 mg/kg AA, a subsequent study was conducted with four experimental groups (n = 6): Control (non-irradiated), TLI, TLI + AA (100 mg/kg), and TLI + AA + T0070907. The PPARγ antagonist T0070907 (Abmole, China) was prepared in vehicle solution (5% DMSO + 45% PEG 300 + H₂O) and administered via intraperitoneal injection at 1 mg/kg twice daily throughout the 14-day treatment period. AA was administered as described above, with all treatments initiated 24 hours post-irradiation. Tissues were collected 21 days after irradiation for subsequent analysis.

### Collection of fecal samples from donors and preparation of fecal suspensions

Healthy 6- to 8-week-old C57BL/6J mice from the Control group, maintained under specific pathogen-free conditions, were selected as FMT donors based on stable body weight, glossy fur, and normal activity. Donors were transiently placed in sterilized cages, and fresh feces were aseptically collected using sterile forceps into nuclease-free 2 mL tubes. Fecal suspensions were prepared according to modified established protocols [52,55]. Briefly, samples were homogenized by vortexing in reduced physiological saline at 0.1 g feces/mL to preserve microbial viability, followed by homogenized for 10 min. After centrifugation at 3,000 rpm (4°C, 1 min) to remove insoluble debris, the suspensions were aliquoted, flash-frozen in liquid nitrogen, and stored at -80°C until daily gavage administration.

### 16S rRNA sequencing

Fresh fecal samples were collected under aseptic conditions, immediately flash-frozen in liquid nitrogen, and stored at -80°C until further processing. Bacterial DNA was extracted using the TIANamp Stool DNA Kit (TIANGEN, DP328, China), with modifications for improved yield, including bead-beating for 5 minutes. DNA quality was assessed by NanoDrop spectrophotometer and agarose gel electrophoresis. The V3-V4 and V4-V5 regions of the 16S rRNA gene were amplified using specific primers. PCR was conducted using conventional thermal cycling conditions. Amplicons were purified using AMPure XP beads (Beckman Coulter, USA). Libraries were constructed, quantified, and sequenced on the NovaSeq 6000 platform (Illumina) with PE250 configuration. Raw sequences were processed using QIIME2 (https://qiime2.org/), including merging, quality filtering (Phred score > 20), and chimera removal with DADA2. Reads were clustered into OTUs at 97% similarity or assigned as ASVs at 100% similarity. Alpha diversity metrics (Observed features, Chao1, Simpson, Dominance indices) were calculated, and taxonomic classification was performed to assess microbial composition. Volcano plots visualized differentially abundant taxa at the genus level. Statistical analysis was conducted using R software (4.2.0).

### LC-MS untargeted metabolomics

First, fresh fecal samples were aseptically collected, flash-frozen in liquid nitrogen, and stored at -80°C. The samples were then homogenized and mixed with pre-chilled 80% methanol, followed by thorough vortexing to ensure complete extraction. Next, the samples were thawed on ice and vortexed for 30 seconds, then subjected to 6 minutes of sonication to facilitate cell disruption and metabolite release. The processed samples were centrifuged at 5,000 rpm for 1 minute at 4°C, and the supernatant was collected. The supernatant was freeze-dried and re-dissolved in 10% methanol. The resulting solution was analyzed using a Vanquish UHPLC system coupled with an Orbitrap Q Exactive HF mass spectrometer (Thermo Fisher, USA). During the analysis, linear gradient separation was performed using a Hypersil Gold column. Data were collected by the mass spectrometer for subsequent quantification and identification, ultimately revealing the metabolite composition of the fecal samples. Kyoto Encyclopedia of Genes and Genomes (KEGG) pathway enrichment analysis of differential metabolites and Metabolite Set Enrichment Analysis (MSEA) of all annotated metabolites were performed using the clusterProfiler package in R (4.2.0).

### Transcriptome sequencing

Total RNA was extracted from mouse lung tissue using TRIzol (Invitrogen, USA), and its quality and concentration were assessed with a NanoDrop spectrophotometer (Thermo Fisher, USA) and an Agilent 2100 Bioanalyzer, ensuring RIN > 7. High-quality RNA was used to prepare libraries with the MGIEasy RNA Library Kit (BGI, China), quantified by Qubit and Bioanalyzer (Agilent Technologies, USA), and sequenced on the BGISEQ-500 platform (BGI, China) with a paired-end 150 bp (PE150) configuration, generating ~40 million reads per sample. Raw sequencing reads underwent quality control using FastQC, followed by trimming of low-quality bases and adapter sequences using Trimmomatic. Clean reads were aligned to the reference genome using HISAT2, and gene expression levels were quantified using StringTie. Differential expression was analyzed using DESeq2 (FDR < 0.05), with functional enrichment analysis for biological interpretation.

### Irradiation study

The X-ray irradiation device (X-RAD320iX, USA) was used for both in vivo and in vitro experiments. For whole-lung irradiation in mice, a total dose of 20 Gy was delivered at a dose rate of 2.0 Gy/minutes. Prior to irradiation, mice were deeply anesthetized via pentobarbital sodium, and lead shielding was used to protect non-target areas from radiation exposure. Partial apical lung irradiation and unshielded upper abdominal irradiation (simultaneous exposure of lung and upper abdomen) were administered at the same dose and dose rate, with collimators or shielding plates precisely confining radiation to predefined fields. For cellular experiments, a total dose of 6 Gy was administered at a dose rate of 1.0 Gy/minutes.

### BALF extraction

Bronchoalveolar lavage fluid was collected by rinsing the airways three times with 1 mL of sodium chloride solution through intratracheal injection. The retrieved BALF was centrifuged at 500 × g for 5 minutes at 4°C to separate cellular components from the supernatant. The cell-free supernatant was used for cytokine detection.

### Measurement of MDA and GSH in lung tissue

Equal weights of lung tissue samples were processed for subsequent procedures strictly adhering to the kit instructions of MDA and GSH (Solarbio, China). Absorbance measurements were obtained using the microplate reader. Absorbance was measured using the microplate reader (BioTek Epoch, USA). Data were normalized to total protein content prior to statistical analysis.

### Measurement of lung wet/dry weight ratio

Fresh lung tissue from the right superior lobe was used and rinsed with ice-cold PBS. Surface moisture was blotted away using filter paper. The wet weight of the lung tissue was accurately measured with a precision balance. The samples were then dehydrated at 60°C for 48 hours until a constant mass was achieved to determine the dry weight. The wet-to-dry weight ratio was then calculated based on the obtained measurements.

### Intestinal motility analysis

Mice were orally administered a 100 μL oral dose of 50 mg/mL FITC-dextran (Sigma-Aldrich, USA) in 0.9% PBS. 1 hour later, the mice were euthanized, and the entire gut tract was excised and imaged using the IVIS Spectrum chemiluminescence detection system (Caliper, USA).

### Histological staining

Appropriate amounts of fresh lung tissue were dehydrated, embedded in paraffin, and sectioned into 5 μm slices. After dewaxing, the paraffin sections were stained according to the instructions provided by the HE staining solution (Servicebio, China), Masson’s Trichrome Staining Kit (Servicebio, China), or the Modified Sirius Red Staining Kit (Servicebio, China), respectively. For AB-PAS staining, gut tissue was stained using the AB-PAS staining solution (Servicebio, China). Imaging was performed using an inverted brightfield microscope (Leica, Germany).

Based on established histopathological assessment protocols from previous investigations, lung injury severity was quantitatively evaluated through microscopic analysis of two key parameters: the degree of inflammatory cell infiltration and the extent of alveolar structural damage within pulmonary tissues, while pulmonary fibrosis progression was assessed using the well-validated Ashcroft semi-quantitative scoring system, which provides objective measurement of fibrotic lesion distribution and collagen fiber deposition intensity on a scale of 0–8 (0: normal lung architecture; 1: minimal fibrous thickening of alveolar or bronchiolar walls; 2–3: moderate thickening without obvious damage to lung structure; 4–5: increased fibrosis with definite damage to lung structure and formation of fibrous bands; 6–7: severe distortion of structure with large fibrous areas; 8: total fibrous obliteration of the field) [56,57].

### Colony distribution assessment of fecal microbiota

Fresh fecal samples were collected from different experimental groups of mice under sterile conditions. Following quantification, the samples were serially diluted in sterile PBS to a uniform concentration (1 mg/ml). Aliquots (20 μL) were spread-plated on Luria-Bertani (LB) agar. The plates were incubated at 37°C overnight to allow for bacterial growth. Subsequently, the distribution of single colonies on each plate was assessed.

### Transmission electron microscopy for endoplasmic reticulum morphology analysis

Fresh lung tissue (1 mm^3^) was immediately fixed in 4°C electron microscopy fixative for 2–4 hours. Cell pellets were centrifuged, medium discarded, and fixed at 4°C for 2–4 hours. Samples were rinsed with PBS three times. Post-fixation was performed in 1% osmium tetroxide in 0.1M PBS at room temperature for 2 hours, followed by three PBS rinses. Tissues were dehydrated in a graded ethanol series, each for 15 minutes. Samples were infiltrated with a 1:1 acetone/812 resin mixture overnight, followed by pure 812 resin infiltration overnight, and polymerized at 60°C for 48 hours. Ultra-thin sections (60–80 nm) were double-stained with uranyl acetate and lead citrate (15 minutes each), air-dried, and observed under a transmission electron microscope (FEI, TECNAI G2 TWIN) for endoplasmic reticulum morphology analysis.

### Quantitative real-time PCR (qRT-PCR)

Samples were homogenized in TRIzol, followed by chloroform extraction and centrifugation to collect the supernatant. RNA was precipitated with isopropanol, washed with 75% ethanol. Its concentration and purity were measured using a NanoDrop 2000 spectrophotometer (Thermo Scientific, USA). cDNA synthesis was performed with the PrimeScript RT reagent kit (Takara-Bio, Japan) following the manufacturer’s protocol. qRT-PCR was conducted using SYBR Fluorescence Quantitation Kit (TaKaRa, Japan). Primer sequences are provided in S1 Table.

### Western blotting

Total protein extracts were obtained using RIPA (Beyotime, China). Equal quantities of protein for each sample were separated by SDS-PAGE, and subsequently transferred onto PVDF membranes, and incubated with primary antibodies for immunodetection, followed by secondary antibodies for chemiluminescent detection using Pierce ECL kit (Thermo Fisher, USA). Details of the antibodies used in this study are provided in S2 Table.

### Cell culture

Beas-2B and MLE-12 cells were obtained from the Cell Bank of Type Culture Collection at the Chinese Academy of Sciences (Shanghai, China) and were certified to be mycoplasma-free. The cells were cultured in Dulbecco’s Modified Eagle Medium (Gibco, USA), supplemented with 10% fetal bovine serum (Gemini, USA) and 1% penicillin-streptomycin solution. Cultures were maintained at 37°C in a humidified incubator with 5% CO₂. The treatment concentration of 0.5 μM AA was used for *in vitro* experiments.

### Immunofluorescence

For intracellular staining, cells were fixed with 4% paraformaldehyde (Solarbio, China), and treated with 0.5% Triton X-100 (Sigma, USA) for permeabilization. After blocking with Quick Blocker (Beyotime, China) for 15 minutes, they were incubated overnight at 4°C with specific primary antibodies. Following PBS washes, cells were incubated with fluorescently labeled secondary antibody (Servicebio, China) for 1 hours. DAPI-containing mounting medium was applied, and samples were stored at 4°C. Imaging was performed using inverted fluorescence microscope (Leica, Germany).

### Co-localization analysis of endoplasmic reticulum and lysosomes

To analyze the co-localization of the ER and lysosomes, the cells were incubated with ER-Tracker (Beyotime, China) and LysoTracker (Beyotime, China) according to the manufacturer’s instructions, followed by washing with PBS and staining with Hoechst 33342 for 10 minutes. Images were acquired using a confocal microscope (Nikon, Japan).

### Live/dead cell staining

To assess cell viability, a live/dead staining assay was performed using the Calcein-AM/PI Double Stain Kit (Beyotime, China). Briefly, cells were seeded in a 6-well plate and treated as required by the experimental protocol. After treatment, cells were washed with phosphate-buffered saline (PBS) and incubated with a mixture of Calcein-AM (to stain live cells) and propidium iodide (PI, to stain dead cells) at 37°C for 30 minutes in the dark. Following incubation, cells were washed with PBS to remove excess dye. Fluorescence imaging was conducted using a live cell imaging system (Cytation 3, BioTek, USA) to distinguish live cells (green fluorescence) from dead cells (red fluorescence).

### CCK-8 assay

Cell viability were assessed using the Cell Counting Kit-8 (Bioss, China) according to the manufacturer’s instructions. Cells were seeded in 96-well plates at a density of 2 × 10^3^ cells per well and treated with different doses of AA according to the experimental protocol. Following the corresponding treatment, 10 μL of CCK-8 solution was added to each well, followed by incubation at 37°C for 2 hours. Absorbance was measured at 450 nm using the microplate reader (BioTek Epoch, USA). Relative cell viability was expressed as a percentage of the control group.

### Enzyme-linked immunosorbent assay (ELISA)

The centrifuged serum and BALF samples were used to detect the levels of cytokines by ELISA method. The supernatants were collected and analyzed for protein levels according to the manufacturer’s instructions. ELISA kits for TNF-α (AE90301Mu), IL-6 (AE90247Mu) were purchased from AMEKO (China) and IL-1β (mle098416-J), IL-4 (mle064310-J), IL-5 (mle063157-J) and IFN-γ (ml002277) were purchased from Mlbio (China). Optical density was measured at 450 nm using the microplate reader (BioTek Epoch, USA).

### Dual-luciferase reporter assay

HEK293T cells were transfected with Lipofectamine 2000 (Invitrogen, USA) using pGL4.74 (0.4 μg) as the backbone reporter plasmid, alongside either pcDNA3.1 or pcDNA3.1-PPARγ (0.4 μg) and either pGL4.20-FAM134B-WT or pGL4.20-FAM134B-Mut (0.4 μg). After 48 hours of transfection, Renilla and Firefly luciferase activities were measured with the Synergy Mx Multi-Mode Microplate Reader (BioTek, USA), and relative luciferase activity was determined by normalizing Firefly signals to Renilla.

### ChIP-qPCR

The ChIP assay was performed using the ChIP kit from Beyotime (China). Chromatin was crosslinked with 1% formaldehyde for 10 minutes, washed with PBS, and lysed in the buffer containing 0.5% SDS, 10 mM EDTA, and 50 mM Tris-HCl. The chromatin was fragmented by sonication and incubated overnight with specific antibodies. Immunoprecipitation was performed using Protein A/G magnetic beads. After reversing the crosslinks, DNA was treated with proteinase K and RNase A, followed by phenol/chloroform extraction and purification. The purified DNA was analyzed by qPCR using SYBR Green PCR Master Mix (YEASEN, China) and gene-specific primers. The enrichment of the target gene was calculated by comparing it with the input DNA.

### Redundancy analysis

Redundancy analysis (RDA) was performed using the microbiota abundance as independent variables and the metabolite concentrations as dependent variables. RDA analysis was performed using the vegan package in R (4.2.0) to explore the role of microbiota in shaping host metabolism.

### Correlation network

Correlation network analyses were performed using R (4.2.0) with the ggplot2 and linkET packages. Initially, uploaded data were cleaned to remove inconsistencies and ensure accuracy. Following data cleaning, pairwise correlation analyses were conducted to explore relationships between variables. Finally, the results were visualized through correlation heatmaps and network plots to illustrate significant associations.

### Molecular docking

The PPARγ protein structure (UniProt ID: P37231) was obtained from UniProt and modeled using AlphaFold. Binding sites on the FAM134B promoter were predicted with Jaspar-scan, selecting the highest-scoring binding sequence. The model was constructed using AlphaFold3, and the best model was selected based on the pLDDT score. Protein-nucleic acid docking was performed using HDOCKlite v1.1, which samples and scores binding modes.

### Statistical analysis

For two-group comparisons, data were analyzed using a two-tailed Student’s t-test or Wilcoxon rank sum test. For comparisons involving more than two groups influenced by a single factor, one-way analysis of variance (ANOVA) followed by Tukey’s post hoc tests, or the Kruskal-Wallis test with Dunn’s correction was applied for multiple comparisons. A *p*-value of < 0.05 was considered statistically significant. Statistical analysis and statistical graphs were conducted using GraphPad Prism 8 or R (4.2.0).

## Supporting information

S1 Fig Serum inflammatory cytokine levels across different experimental mouse groups. (A-E) Serum levels of IL-1β, IL-4, IL-6, TNF-α and IFN-γ in mice from various experimental groups. n = 6 per group. Data are presented as mean ± SD. Statistical comparisons were performed by one-way ANOVA with Tukey’s post hoc test (A-E). * *p* < 0.05, ** *p* < 0.01, *** *p* < 0.001.
(TIF)

S2 Fig Pre-Abx alone does not ameliorate RILI but is required prior to FMT. (A-C) Representative Masson and H&E staining of lung tissues (100× and 400×), along with corresponding quantitative scoring results. n = 6 per group. (D-E) GSH and MDA levels of lung tissue. (F) Lung wet/dry weight ratio across groups. (G) The change of body weight of each experimental mouse. n = 6 per group. Data are presented as mean ± SD. Statistical comparisons were performed by one-way ANOVA with Tukey’s post hoc test (B-F) or two-way ANOVA with Tukey’s post hoc test (G). * *p* < 0.05, ** *p* < 0.01, *** *p* < 0.001.
(TIF)

S3 Fig FMT attenuates pulmonary fibrosis in RILI mice. (A) Representative Masson and HE staining of lung across different groups at 8 weeks post-irradiation. (B-C) Ashcroft fibrosis scores and histopathological lung injury scores in different groups. n = 6 per group. (D) qPCR analysis of *CDH1*, *CDH2*, *ACTA2* and *VIM* mRNA levels. n = 4 per group. (E) Western blot results of EMT markers. Data are presented as mean ± SD. Statistical comparisons were performed using Kruskal-Wallis test with Dunn’s post hoc test (B-C) or one-way ANOVA with Tukey’s post hoc test (D). * *p* < 0.05, ** *p* < 0.01, *** *p* < 0.001.
(TIF)

S4 Fig Precise localization of the irradiated target area in TLI models and systemic gastrointestinal effects induced by distinct thoracic-abdominal radiation exposures. (A) Micro-CT showing the lung irradiation field of TLI models. (B) The visualization of lead shielding for radiation protection of non-target regions. (C) Survival curves post-20 Gy within 14 days across three groups. (D) Body weight changes post-20 Gy across three groups. (E) Intestinal motality test across three groups. (F) Representative images of the intestine under the stereomicroscope across three groups. (G) Intestinal AB-PAS staining across three groups. n = 6 per group.
(TIF)

S5 Fig Representative nutrient agar plate cultures of diluted fecal suspensions from different experimental groups. (TIF)

S6 Fig Relative abundance of specific bacterial taxa across groups in bubble plot. The size of each bubble represents the relative abundance percentage, with colors indicating different treatment groups. n = 6 per group.
(TIF)

S7 Fig MSEA of the arachidonic acid metabolism pathway in different experimental comparisons. (A-C) MSEA plot comparing TLI group vs. control group, TLI + FMT group vs TLI group and TLI + ABS group vs TLI group, showing enrichment of metabolites related to arachidonic acid metabolism.
(TIF)

S8 Fig Serum arachidonic acid levels across different experimental groups. n = 6 per group. Data are presented as mean ± SD. Statistical comparisons were performed by one-way ANOVA with Tukey’s post hoc test. ** *p* < 0.01, *** *p* < 0.001.
(TIF)

S9 Fig Cell viability under irradiation at varying AA concentrations. A dose-dependent experiment was conducted using CCK-8 assays to identify the optimal concentration of AA under 6Gy irradiation. n = 6 per group. Data are presented as mean ± SD. Statistical comparisons between indicated groups were performed using two-sided unpaired Student’s t-tests. * *p* < 0.05, ** *p* < 0.01.
(TIF)

S10 Fig Immunofluorescence of γH2AX foci 12 hours post-AA treatment. Immunofluorescence staining showed γH2AX foci in cells 12 hours after 6Gy irradiation exposure followed by AA treatment, used to assess DNA damage response.
(TIF)

S11 Fig Molecular docking analysis of PPARγ with AA. (A) Overall view of the PPARγ protein (green cartoon) showing the binding pose of arachidonic acid (AA, yellow sticks) within the ligand-binding domain. (B) Close-up view illustrating the key interactions between AA and PPARγ.
(TIF)

S12 Fig Protective effects of different doses of AA supplementation on RILI in mice. (A) Representative Masson and H&E staining of lung tissues (100× and 400×). n = 6 per group. (B-C) GSH and MDA levels of lung tissue. (D) Lung wet/dry weight ratio across groups. (E) The change of body weight of each experimental mouse. n = 6 per group. Data are presented as mean ± SD. Statistical comparisons were performed by one-way ANOVA with Tukey’s post hoc test (B-D) or two-way ANOVA with Tukey’s post hoc test (E). * p < 0.05, ** p < 0.01, *** p < 0.001.
(TIF)

S1 Table The sequences of the qRT-PCR primers and ChIP-qPCR-Primers. (DOCX)

S2 Table A list of all antibodies used in this work and dilutions. (DOCX)

S3 Table Numerical data of this study. Source data for Figs 2C, 2D, 2E, 2F, 2G, 2H, 2I, 2J, 2K, 2L, 3C, 3H, 3I, 3J, 3K, 4C, 4D, 4E, 4F, 4G, 4H, 4I, 4J, 4K, 5D, 8B, 9C, 9D, 10G, 10H, 11C, 11D, 11E, 11F, 11G, 11H, 11I, 11J, S1A, S1B, S1C, S1D, S1E, S2B, S2C, S2D, S2E, S2F, S2G, S3B, S3C, S3D, S4C, S4D, S8, S9, S12B, S12C, S12D, and S12E are provided as an Excel workbook with separate worksheets per panel.
(XLSX)

S1 File Original blots of this study. The original blots for all Western Blot are provided in the file.
(PDF)
