# Supplementary figures and images for: Fecal microbiota transplantation ameliorates radiation-induced lung injury by reshaping gut metabolic homeostasis to activate FAM134B-mediated ER-phagy
Source: PLoS Pathog. 2026 Jan 21;22(1):e1013786. doi: 10.1371/journal.ppat.1013786 (PMC12822986; doi:10.1371/journal.ppat.1013786)

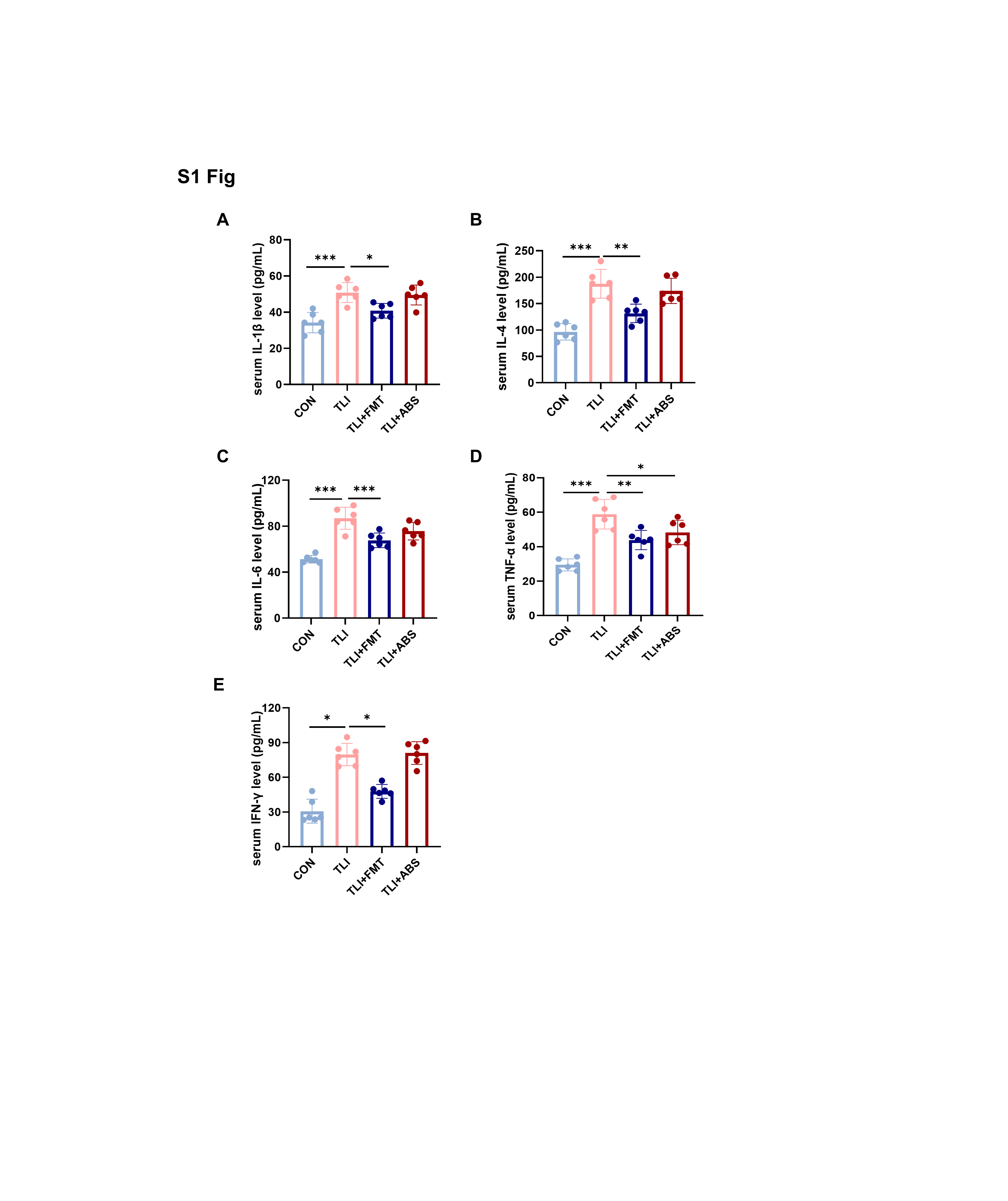

Supplement: S1 Fig — (A-E) Serum levels of IL-1β, IL-4, IL-6, TNF-α and IFN-γ in mice from various experimental groups. n = 6 per group. Data are presented as mean ± SD. Statistical comparisons were performed by one-way ANOVA with Tukey’s post hoc test (A-E). * p < 0.05, ** p < 0.01, *** p < 0.001. (TIF) [file ppat.1013786.s001.TIF]

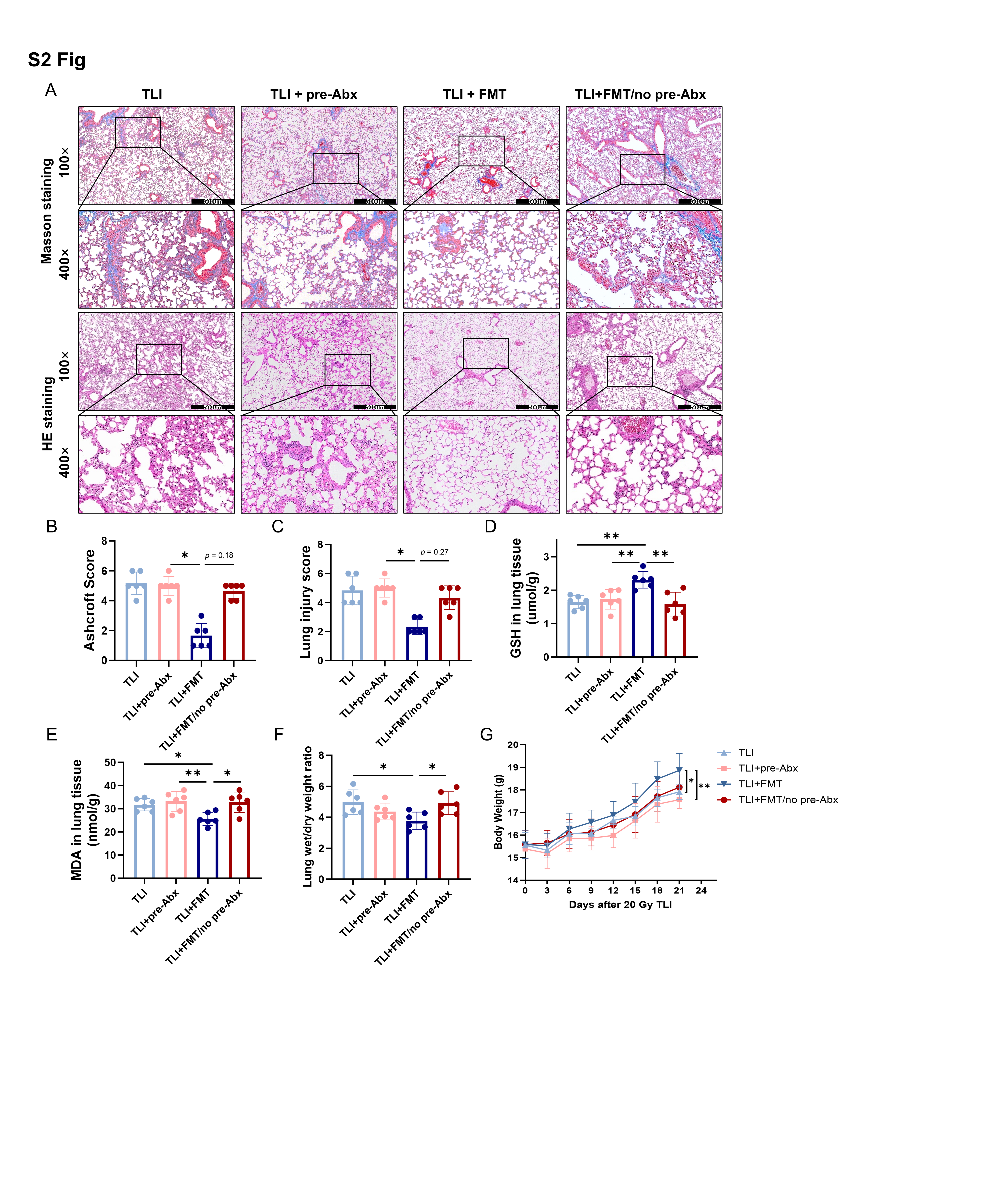

Supplement: S2 Fig — (A-C) Representative Masson and H&E staining of lung tissues (100× and 400×), along with corresponding quantitative scoring results. n = 6 per group. (D-E) GSH and MDA levels of lung tissue. (F) Lung wet/dry weight ratio across groups. (G) The change of body weight of each experimental mouse. n = 6 per group. Data are presented as mean ± SD. Statistical comparisons were performed by one-way ANOVA with Tukey’s post hoc test (B-F) or two-way ANOVA with Tukey’s post hoc test (G). * p < 0.05, ** p < 0.01, *** p < 0.001. (TIF) [file ppat.1013786.s002.TIF]

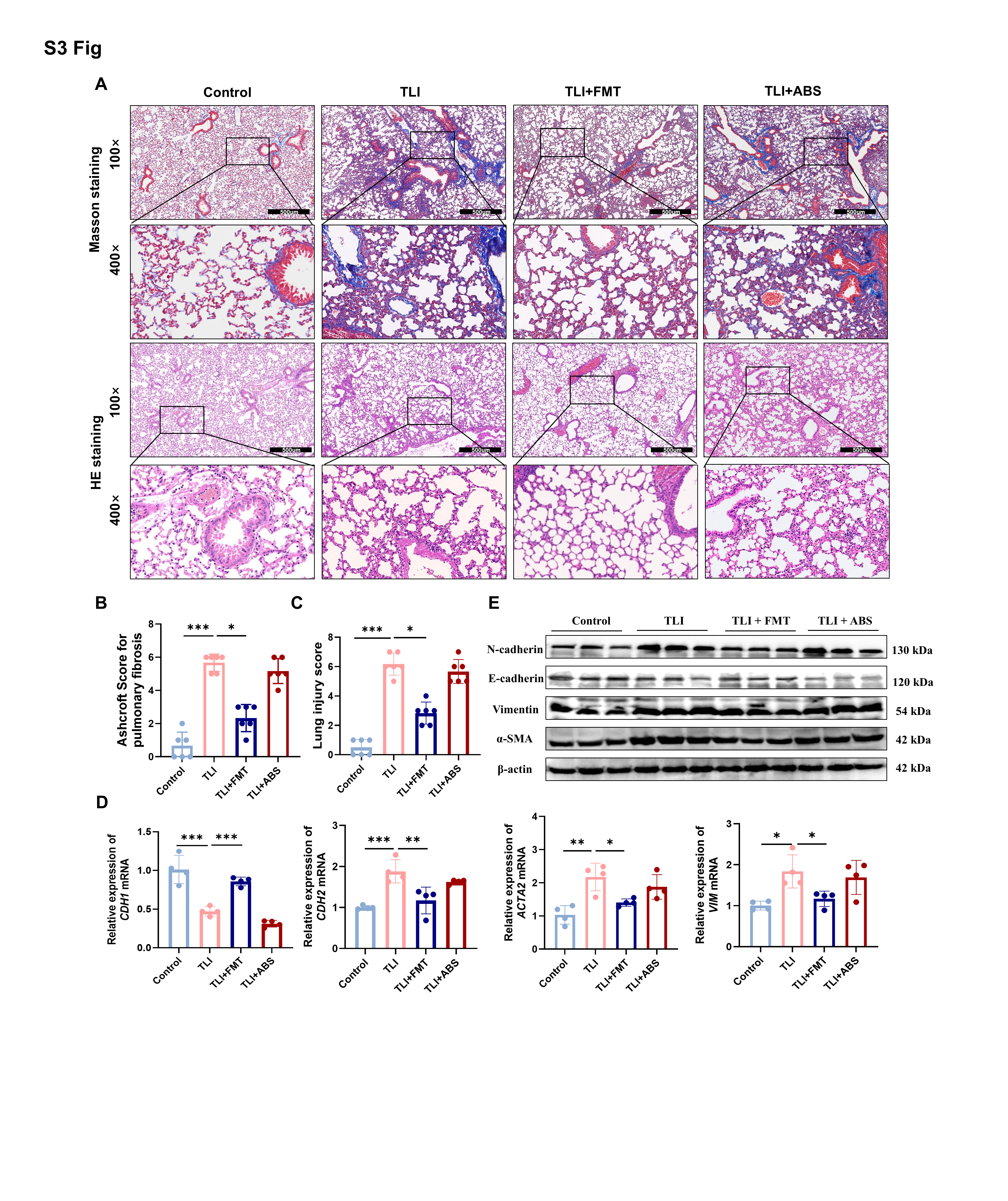

Supplement: S3 Fig — (A) Representative Masson and HE staining of lung across different groups at 8 weeks post-irradiation. (B-C) Ashcroft fibrosis scores and histopathological lung injury scores in different groups. n = 6 per group. (D) qPCR analysis of CDH1, CDH2, ACTA2 and VIM mRNA levels. n = 4 per group. (E) Western blot results of EMT markers. Data are presented as mean ± SD. Statistical comparisons were performed using Kruskal-Wallis test with Dunn’s post hoc test (B-C) or one-way ANOVA with Tukey’s post hoc test (D). * p < 0.05, ** p < 0.01, *** p < 0.001. (TIF) [file ppat.1013786.s003.TIF]

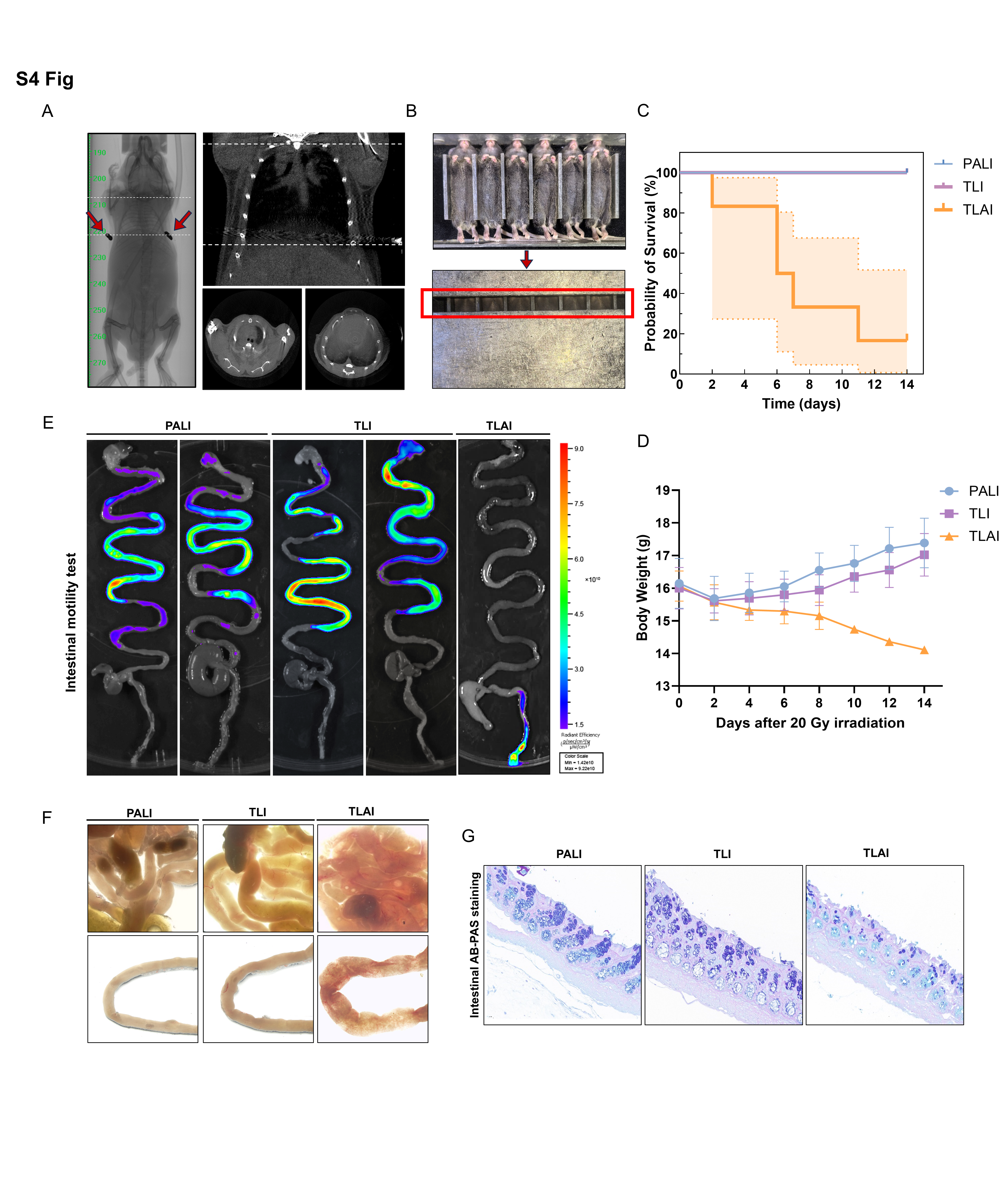

Supplement: S4 Fig — (A) Micro-CT showing the lung irradiation field of TLI models. (B) The visualization of lead shielding for radiation protection of non-target regions. (C) Survival curves post-20 Gy within 14 days across three groups. (D) Body weight changes post-20 Gy across three groups. (E) Intestinal motality test across three groups. (F) Representative images of the intestine under the stereomicroscope across three groups. (G) Intestinal AB-PAS staining across three groups. n = 6 per group. (TIF) [file ppat.1013786.s004.TIF]

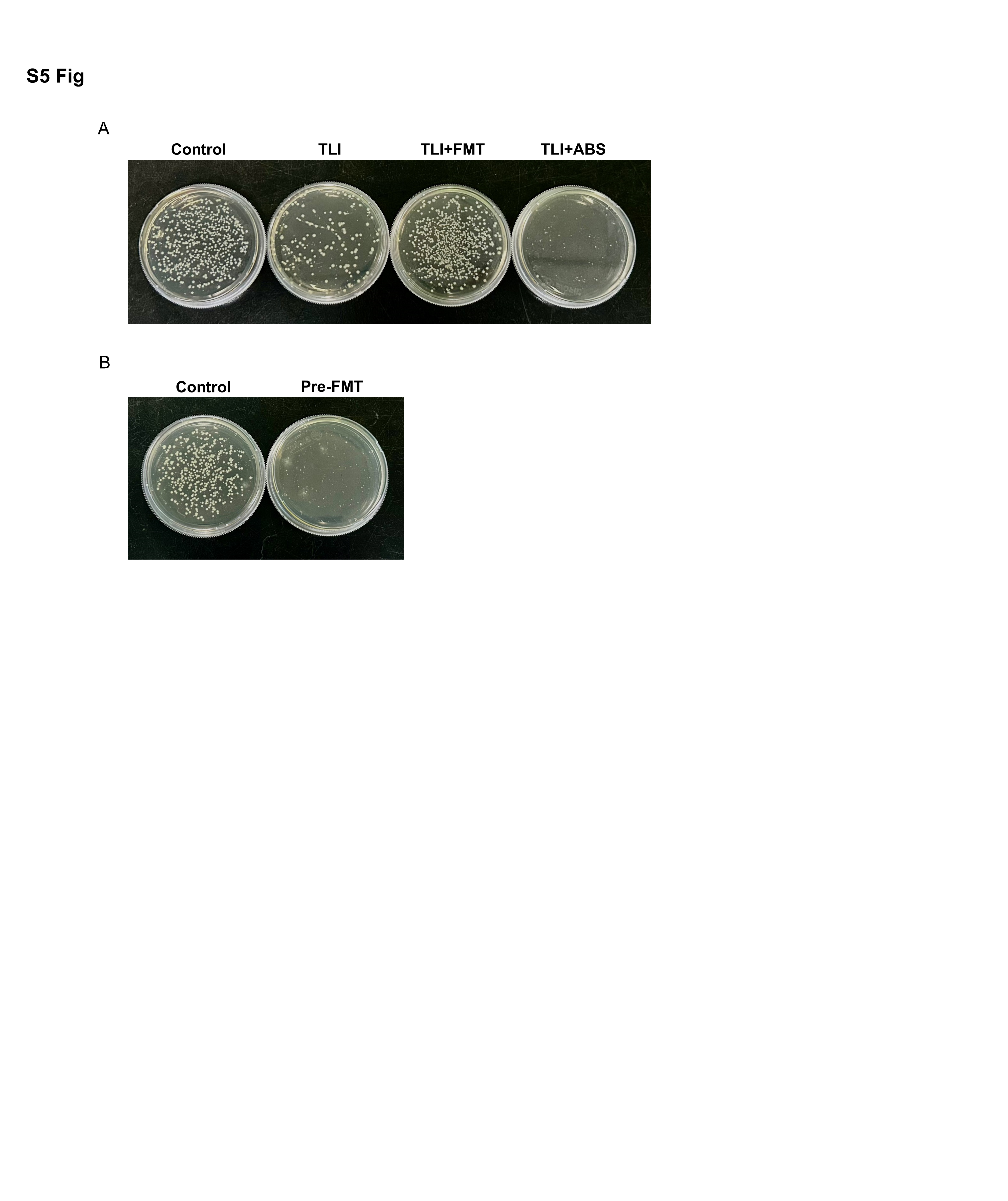

Supplement: S5 Fig — (TIF) [file ppat.1013786.s005.TIF]

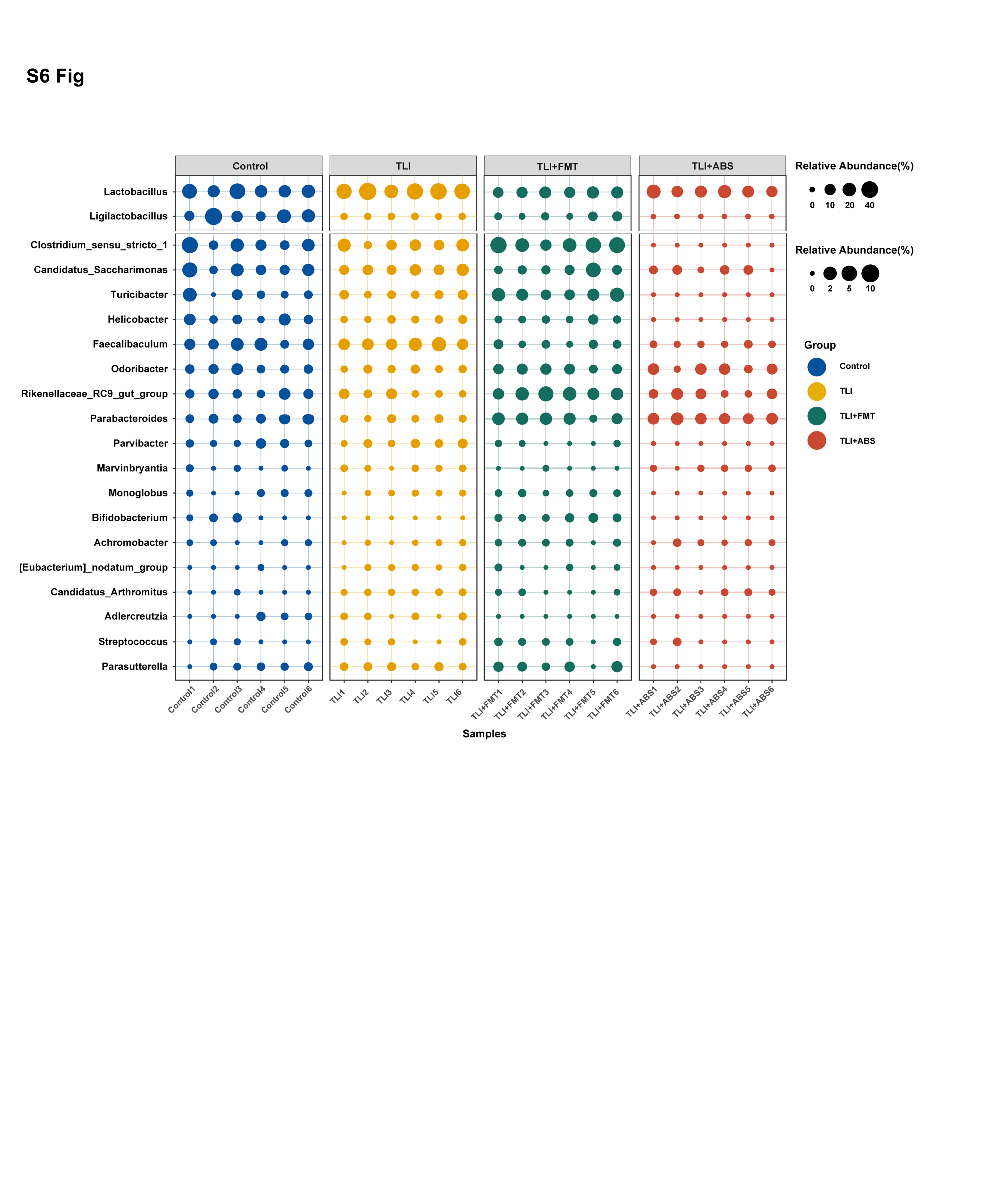

Supplement: S6 Fig — The size of each bubble represents the relative abundance percentage, with colors indicating different treatment groups. n = 6 per group. (TIF) [file ppat.1013786.s006.TIF]

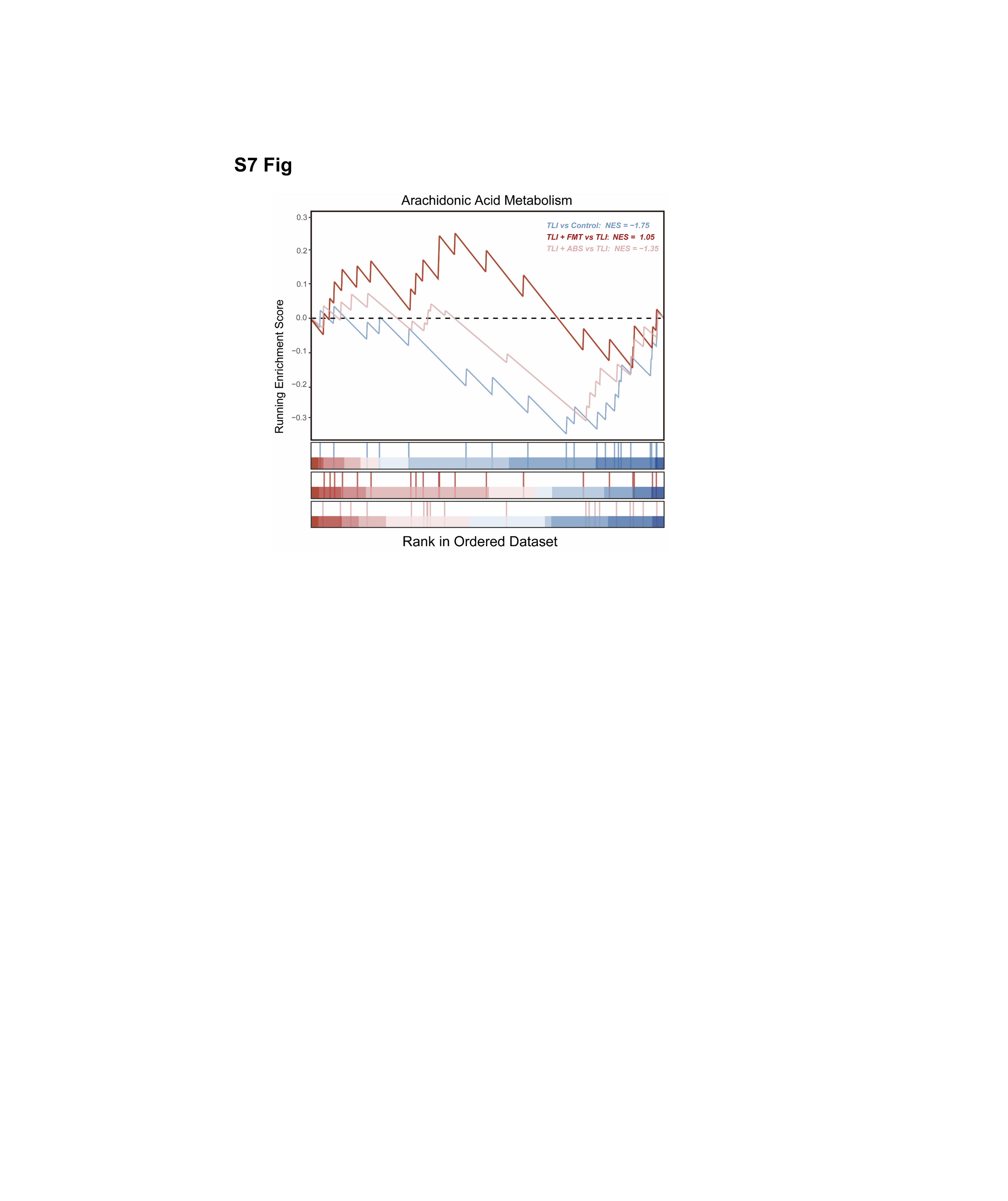

Supplement: S7 Fig — (A-C) MSEA plot comparing TLI group vs. control group, TLI + FMT group vs TLI group and TLI + ABS group vs TLI group, showing enrichment of metabolites related to arachidonic acid metabolism. (TIF) [file ppat.1013786.s007.TIF]

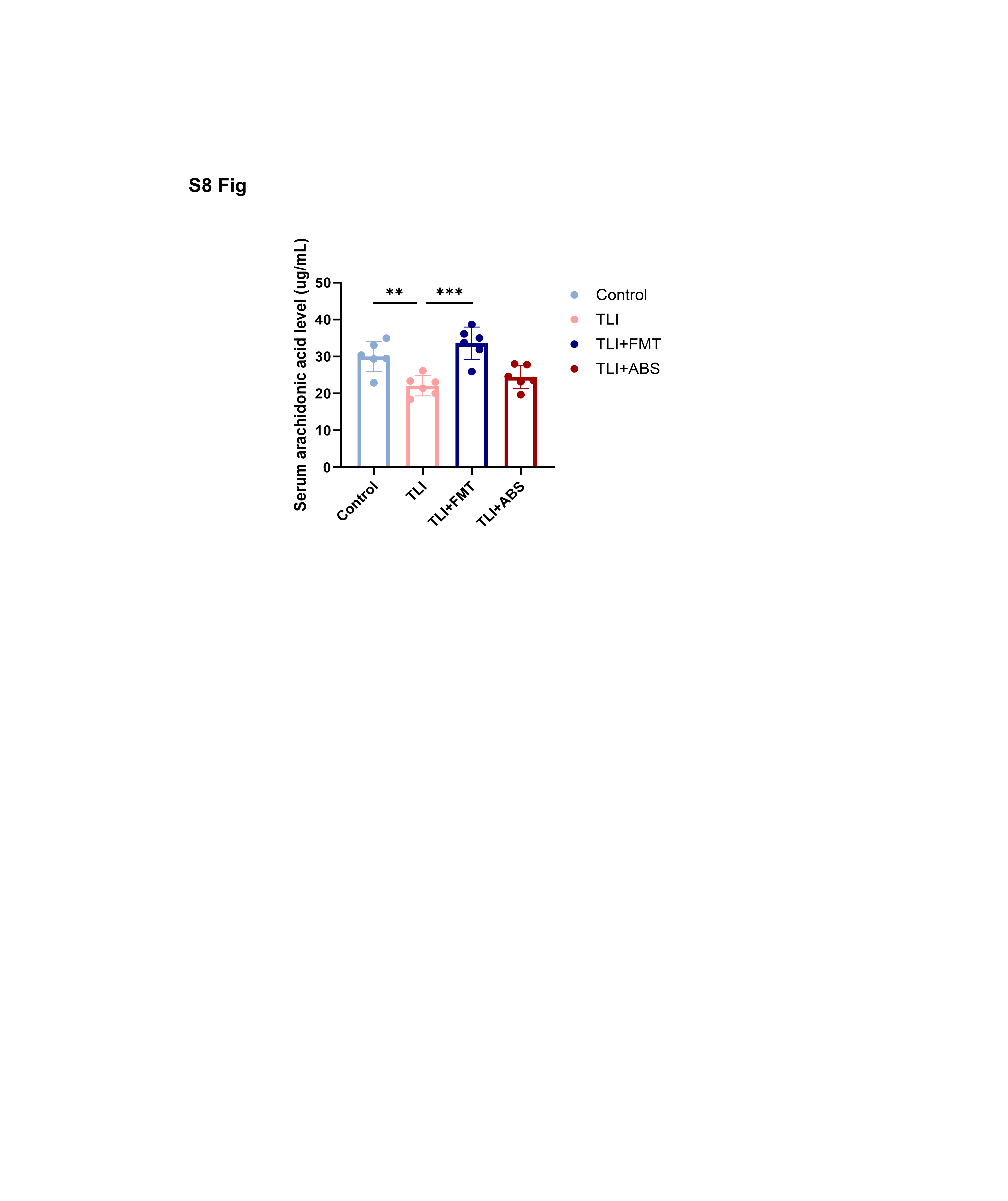

Supplement: S8 Fig — Data are presented as mean ± SD. Statistical comparisons were performed by one-way ANOVA with Tukey’s post hoc test. ** p < 0.01, *** p < 0.001. (TIF) [file ppat.1013786.s008.TIF]

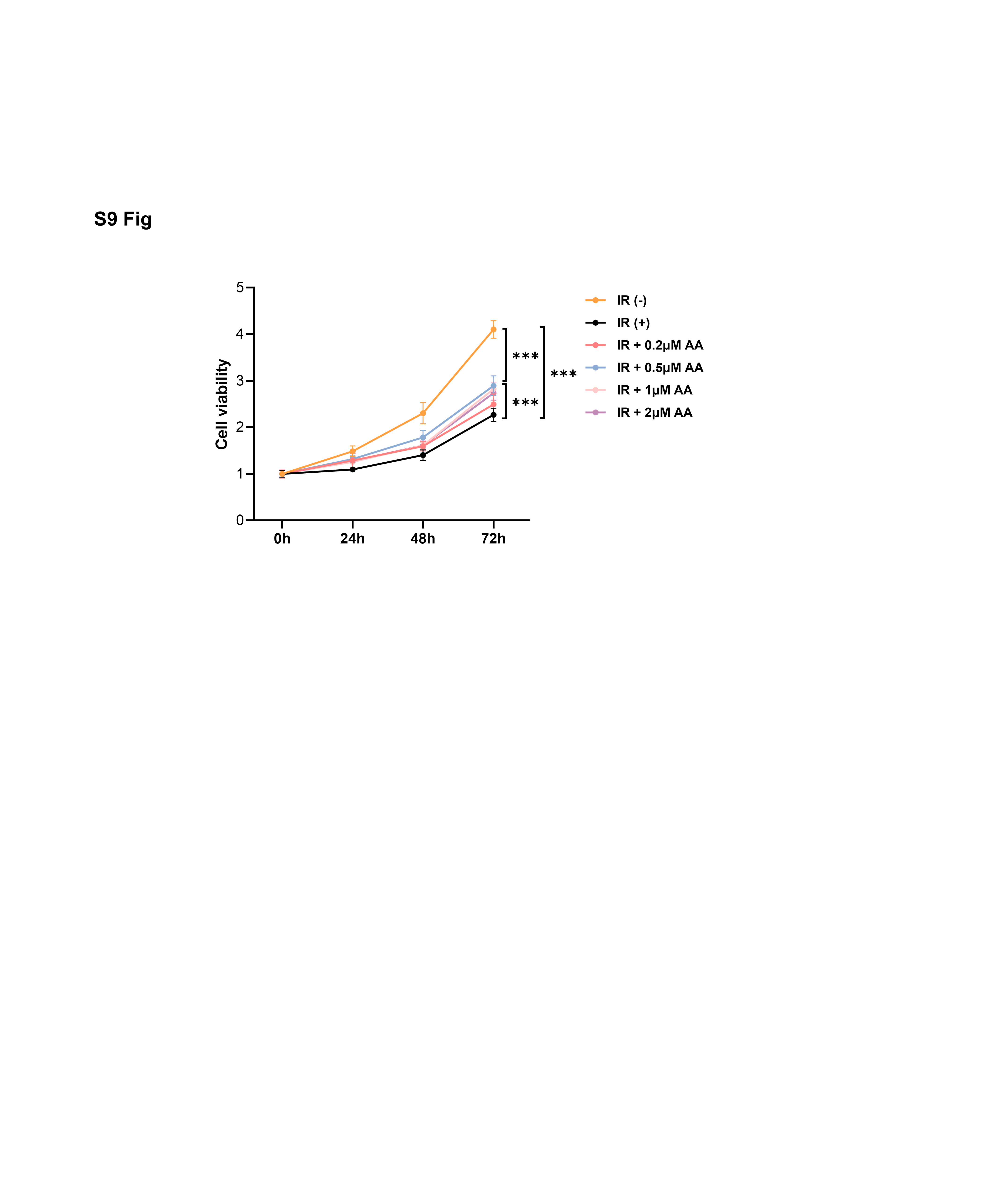

Supplement: S9 Fig — A dose-dependent experiment was conducted using CCK-8 assays to identify the optimal concentration of AA under 6Gy irradiation. n = 6 per group. Data are presented as mean ± SD. Statistical comparisons between indicated groups were performed using two-sided unpaired Student’s t-tests. * p < 0.05, ** p < 0.01. (TIF) [file ppat.1013786.s009.TIF]

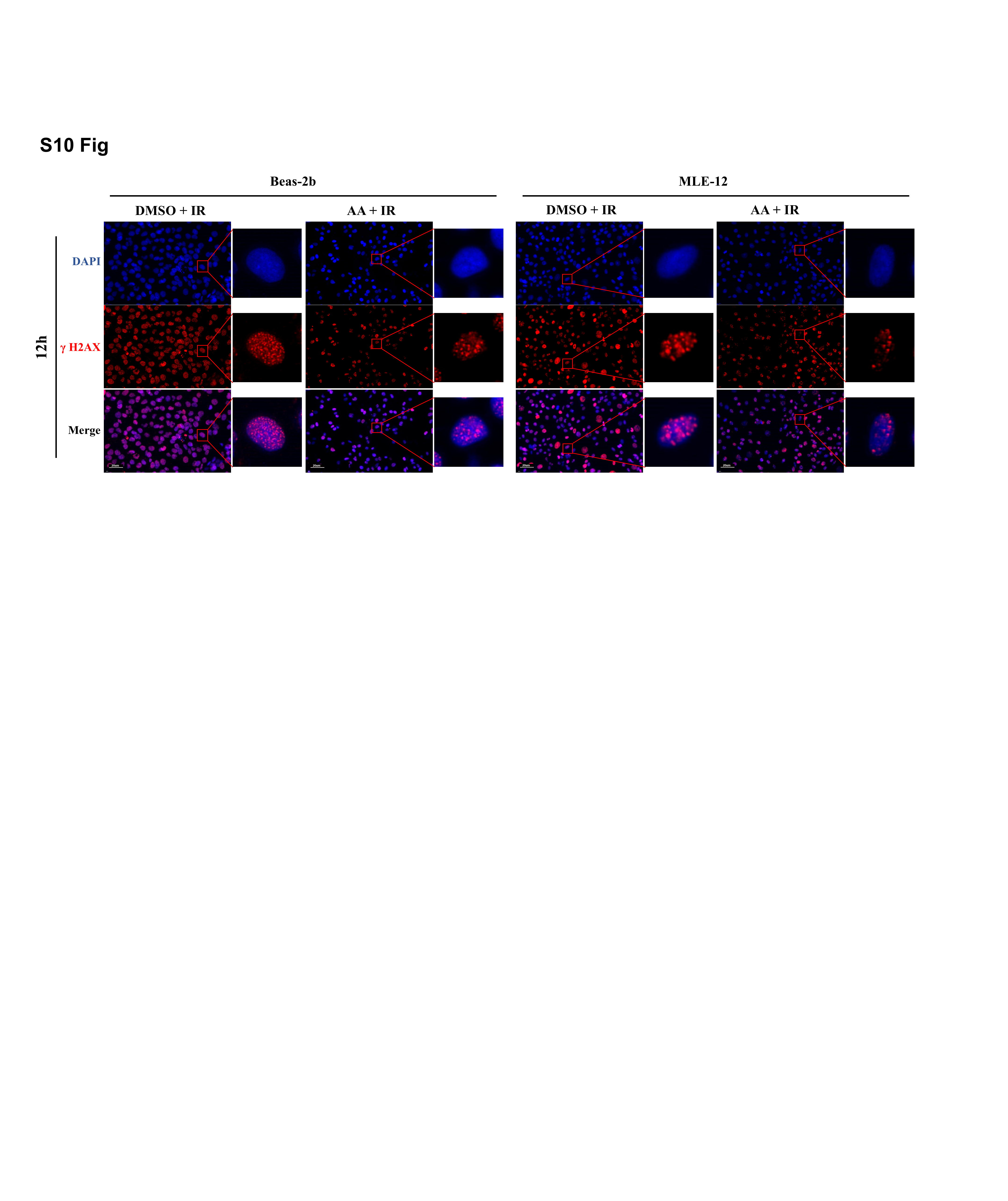

Supplement: S10 Fig — Immunofluorescence staining showed γH2AX foci in cells 12 hours after 6Gy irradiation exposure followed by AA treatment, used to assess DNA damage response. (TIF) [file ppat.1013786.s010.TIF]

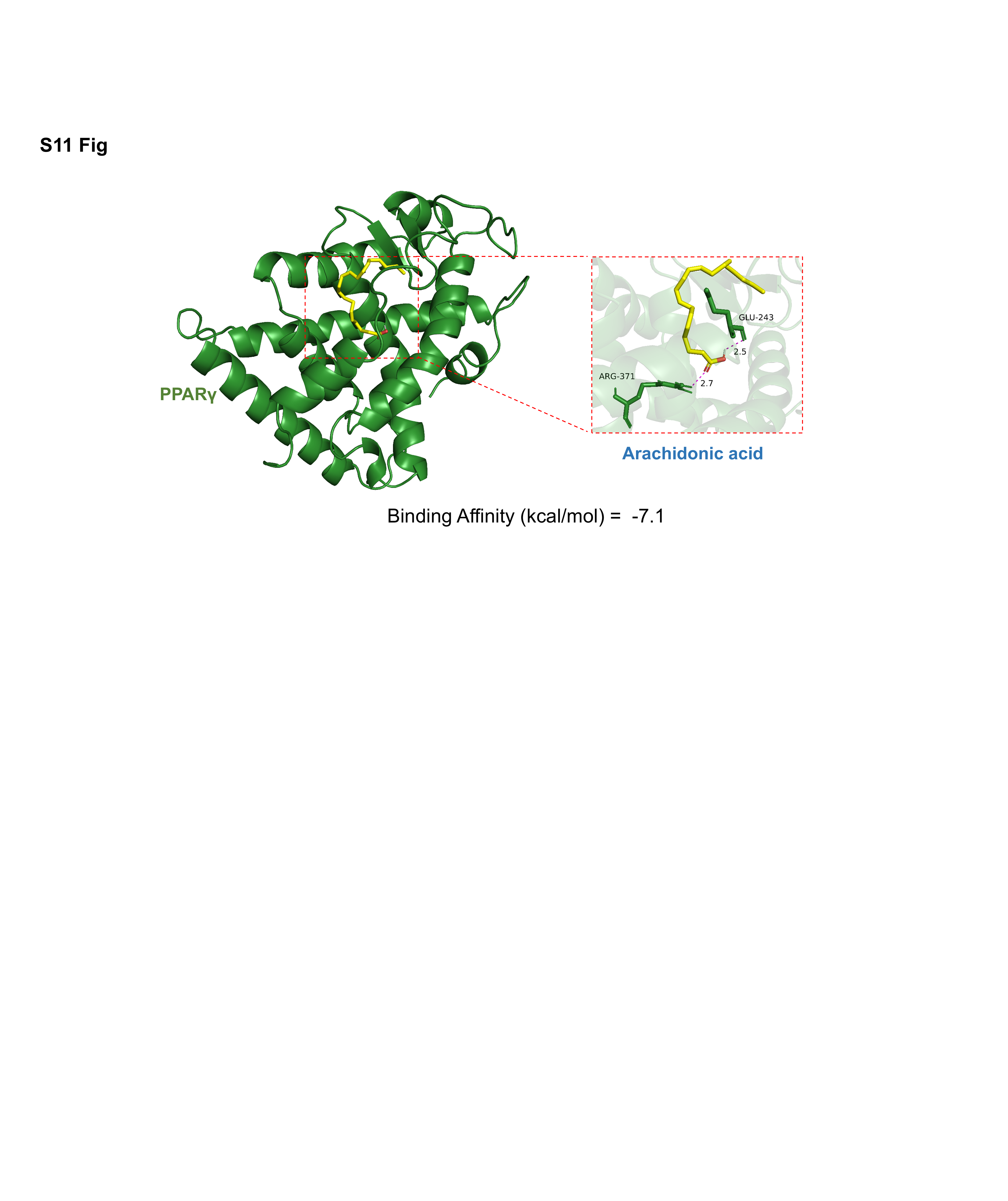

Supplement: S11 Fig — (A) Overall view of the PPARγ protein (green cartoon) showing the binding pose of arachidonic acid (AA, yellow sticks) within the ligand-binding domain. (B) Close-up view illustrating the key interactions between AA and PPARγ. (TIF) [file ppat.1013786.s011.TIF]

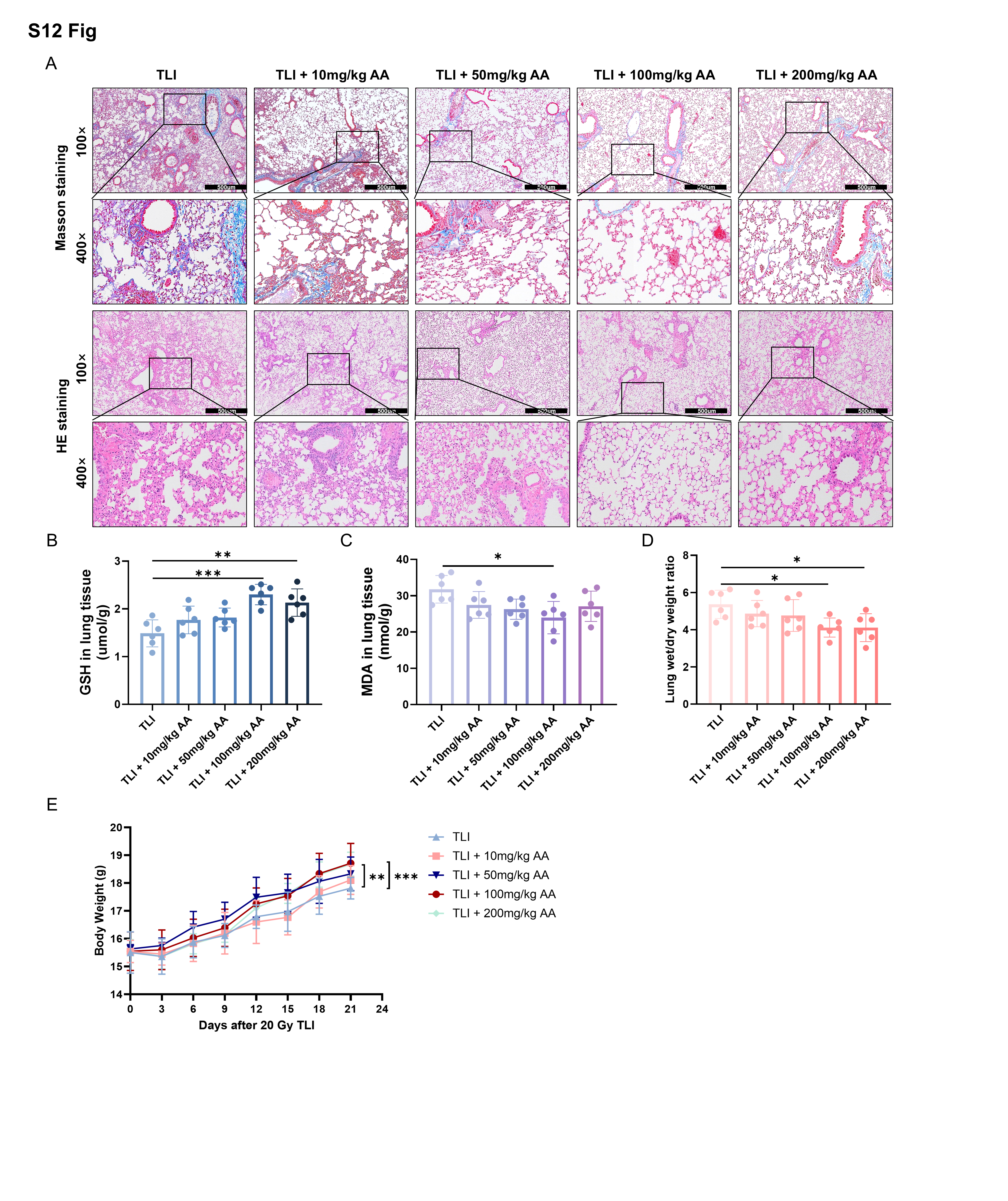

Supplement: S12 Fig — (A) Representative Masson and H&E staining of lung tissues (100× and 400×). n = 6 per group. (B-C) GSH and MDA levels of lung tissue. (D) Lung wet/dry weight ratio across groups. (E) The change of body weight of each experimental mouse. n = 6 per group. Data are presented as mean ± SD. Statistical comparisons were performed by one-way ANOVA with Tukey’s post hoc test (B-D) or two-way ANOVA with Tukey’s post hoc test (E). * p < 0.05, ** p < 0.01, *** p < 0.001. (TIF) [file ppat.1013786.s012.TIF]
