# Supplementary material for: Fecal microbiota transplantation ameliorates radiation-induced lung injury by reshaping gut metabolic homeostasis to activate FAM134B-mediated ER-phagy
Source: PLoS Pathog. 2026 Jan 21;22(1):e1013786. doi: 10.1371/journal.ppat.1013786 (PMC12822986; doi:10.1371/journal.ppat.1013786)
Supplement: S1 Table — (DOCX) [file ppat.1013786.s013.docx]

**S1 Table. The sequences of the qRT-PCR primers and ChIP-qPCR-primers.**

| Primers |  | Sequence 5’ to 3’ |
| --- | --- | --- |
| m-β-actin | Forward | CATCCGTAAAGACCTCTAGCCAAC |
|  | Reverse | ATGGAGCCACCGATCCACA |
| m-CDH1 | Forward | CAGGTCTCCTCATGGCTTTGC |
|  | Reverse | CTTCCGAAAAGAAGGCTGTCC |
| m-CDH2 | Forward | AGCGCAGTCTTACCGAAGG |
|  | Reverse | TCGCTGCTTTCATACTGAACTTT |
| m-VIM | Forward | TGAGATCGCCACCTACAGGA |
|  | Reverse | ACAATGCTTCTCTGGCACGTCT |
| m-ACTA2 | Forward | GGACGTACAACTGGTATTGTGC |
|  | Reverse | TCGGCAGTAGTCACGAAGGA |
| h-GAPDH | Forward | ACGGATTTGGTCGTATTGGG |
|  | Reverse | TGATTTTGGAGGGATCTCGC |
| h-FAM134B-promoter | Forward  Reverse | TTTGGATACGGAACCGAGCA AAACGACCTTTTCCCTTGCAG |
|  |  |  |
