# Supplementary material for: Fecal microbiota transplantation ameliorates radiation-induced lung injury by reshaping gut metabolic homeostasis to activate FAM134B-mediated ER-phagy
Source: PLoS Pathog. 2026 Jan 21;22(1):e1013786. doi: 10.1371/journal.ppat.1013786 (PMC12822986; doi:10.1371/journal.ppat.1013786)
Supplement: S2 Table — (DOCX) [file ppat.1013786.s014.docx]

**S2 Table. A list of antibodies used in this work and dilutions.**

| Antibodies | Catalog number | Company | Dilutions |
| --- | --- | --- | --- |
| Anti-FAM134B | #83414 | CST | 1:1000 |
| Anti-HSPA5 | 66574-1-Ig | Proteintech | 1:5000 |
| Anti-PERK | YT3666 | Immunoway | 1:1000 |
| Anti-ATF6 | YT7559 | Immunoway | 1:1000 |
| Anti-IRE1α | A21021 | Abclonal | 1:1000 |
| Anti-γH2AX | AP0687 | Abclonal | 1:1000 |
| Anti-LC3BI/II | GB113801 | Servicebio | 1:1000 |
| Anti-E-cadherin | BS1098 | Bioworld | 1:1000 |
| Anti-N-cadherin | BS72312 | Bioworld | 1:1000 |
| Anti-Vimentin | YM3158 | Immunoway | 1:1000 |
| Anti-α-SMA | YM3364 | Immunoway | 1:1000 |
| Anti-β-actin | AP0060 | Bioworld | 1:1000 |
| Normal Rabbit IgG | 2729S | CST | 1:100 |
| Goat anti-Mouse IgG (H+L) HRP | BS12478 | Bioworld | 1:50000 |
| Goat anti-Rabbit IgG (H+L) HRP | BS13278 | Bioworld | 1:10000 |
| Cy3 goat anti-Mouse antibody | GB21303 | Servicebio | 1 : 200 |
| Cy3 goat anti-Rabbit antibody | GB21301 | Servicebio | 1 : 200 |
|  |  |  |  |
