# Supplementary material for: Fecal microbiota transplantation ameliorates radiation-induced lung injury by reshaping gut metabolic homeostasis to activate FAM134B-mediated ER-phagy
Source: PLoS Pathog. 2026 Jan 21;22(1):e1013786. doi: 10.1371/journal.ppat.1013786 (PMC12822986; doi:10.1371/journal.ppat.1013786)
Supplement: S1 File — The original blots for all Western Blot are provided in the file. (PDF) [file ppat.1013786.s016.pdf]

Figure 6F

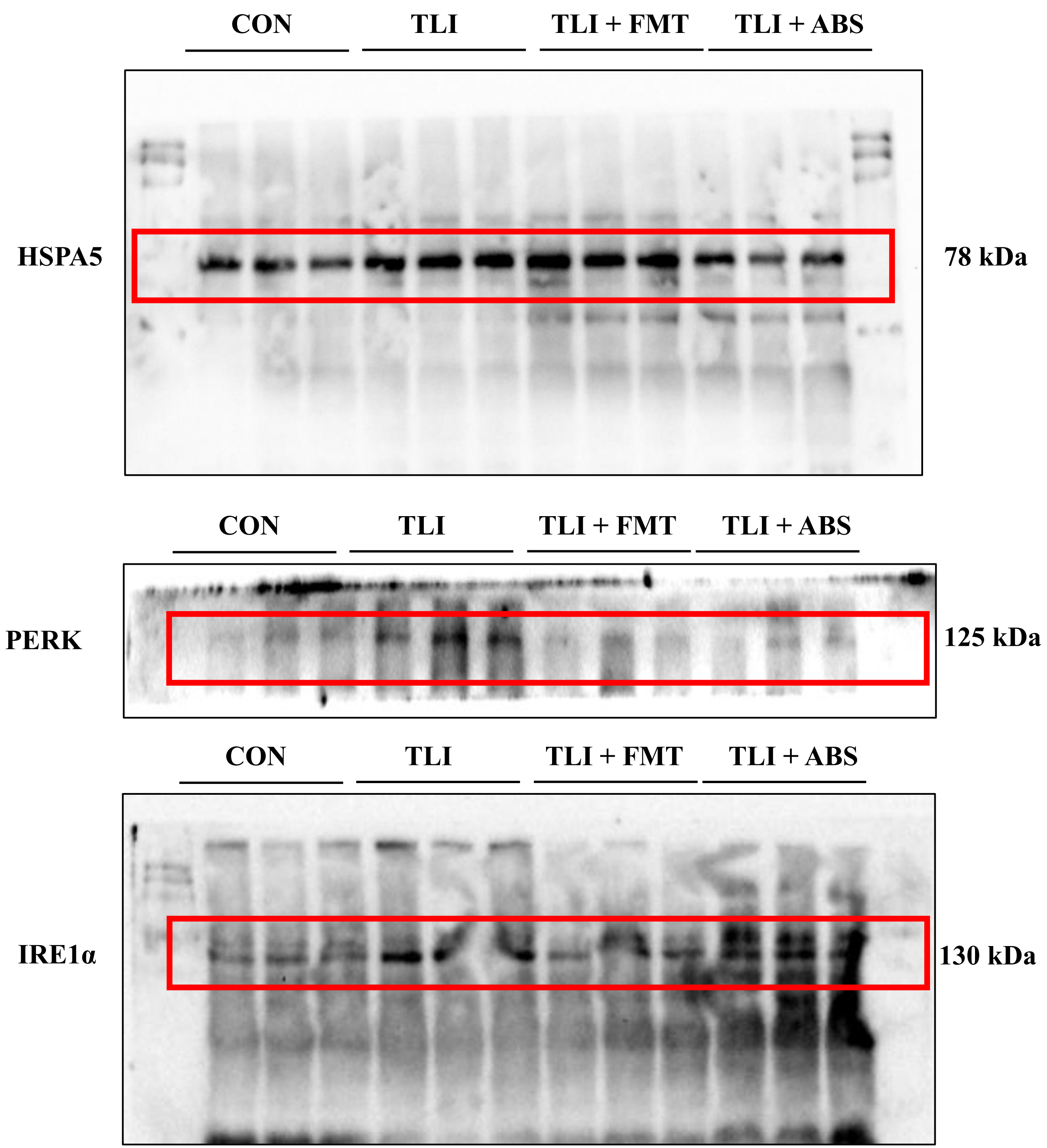
CON
TLI
TLI + FMT
TLI + ABS
HSPA5
78 kDa
CON
TLI
TLI + FMT
TLI + ABS
PERK
125 kDa
CON
TLI
TLI + FMT
TLI + ABS
IRE1α
130 kDa

Figure 6F

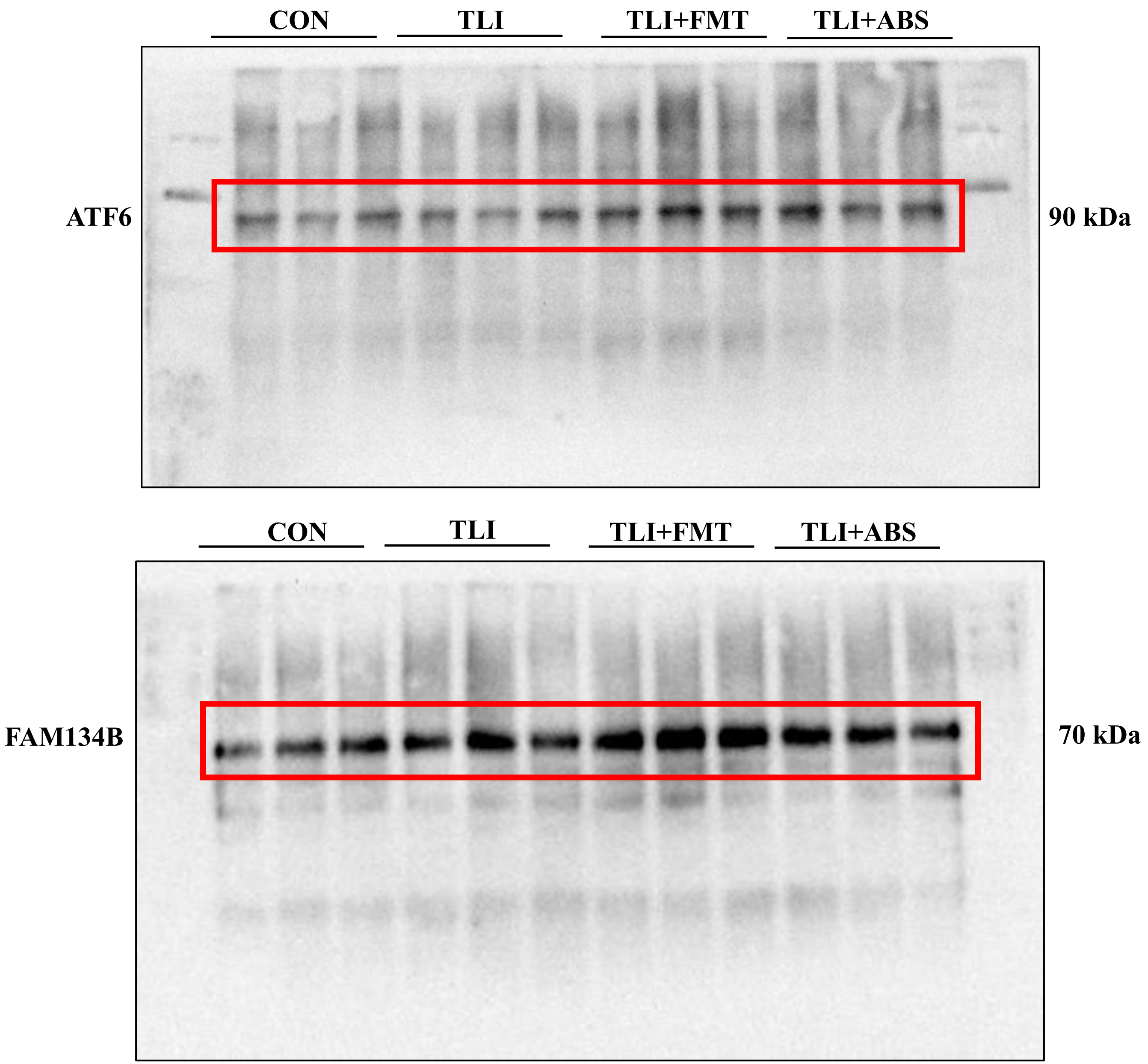

CON
TLI
TLI+FMT
TLI+ABS
ATF6
90 kDa
CON
TLI
TLI+FMT
TLI+ABS
FAM134B
70 kDa

Figure 6F

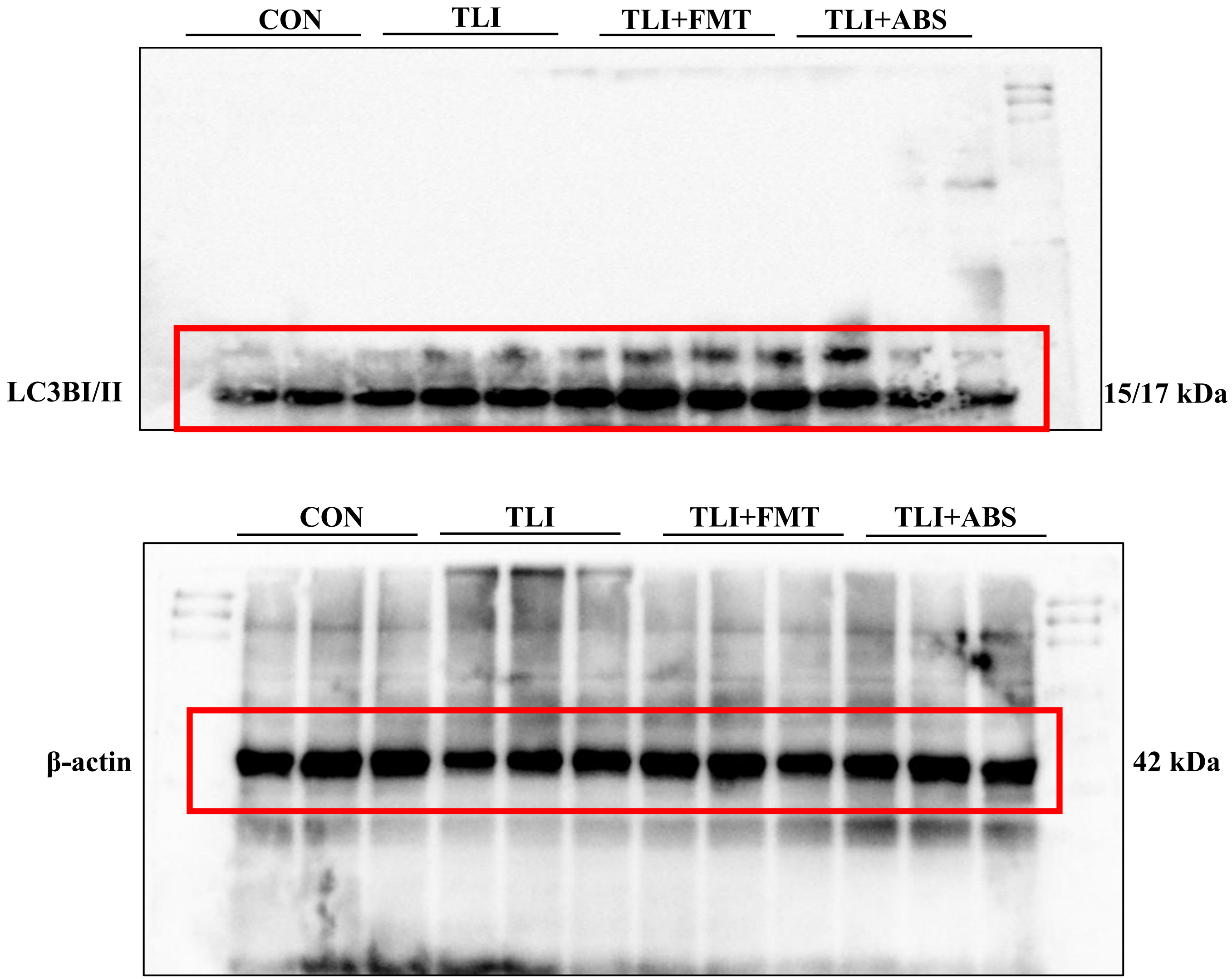

CON
TLI
TLI+FMT
TLI+ABS
LC3BI/II
15/17 kDa
CON
TLI
TLI+FMT
TLI+ABS
β-actin
42 kDa

Figure 8C

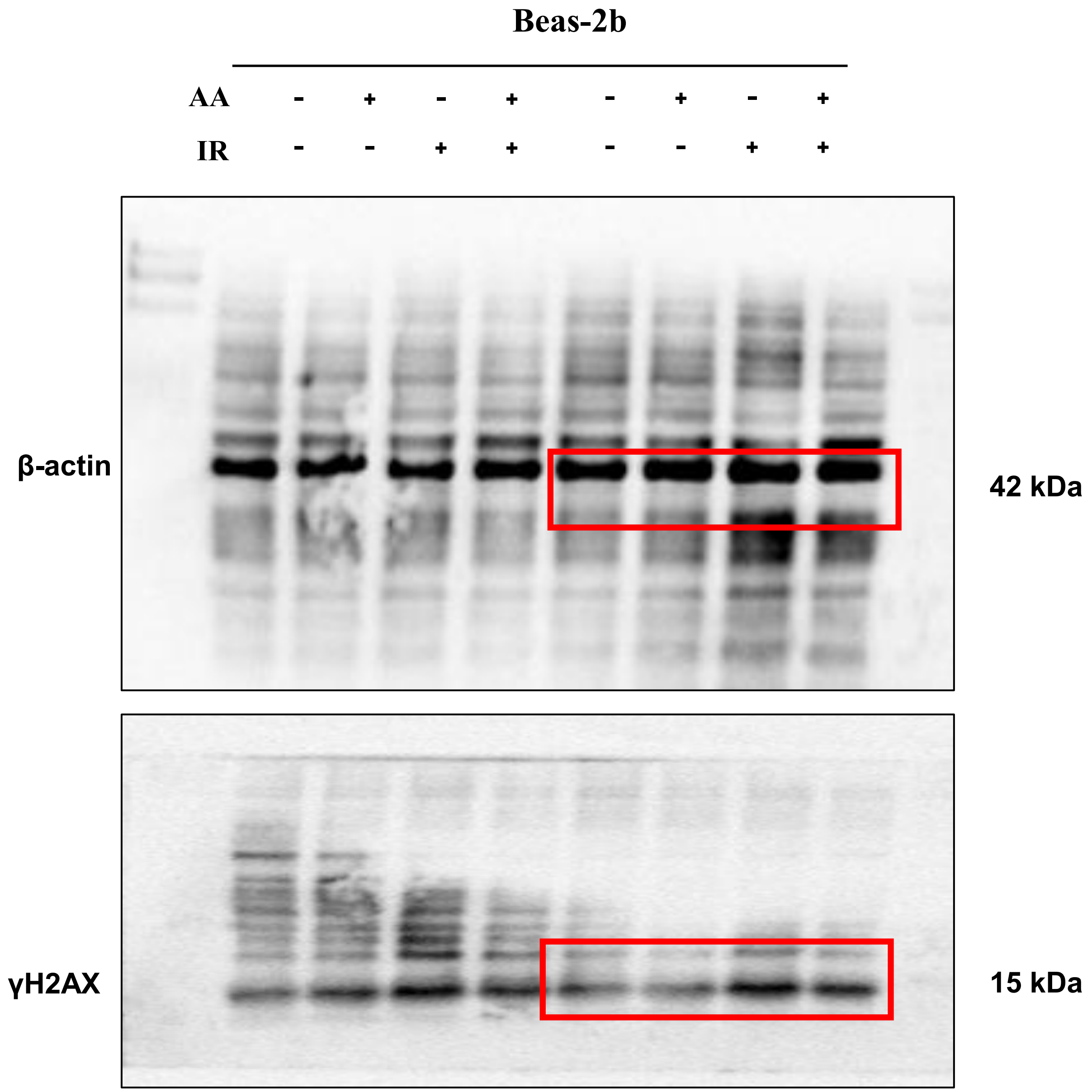

Figure 8C

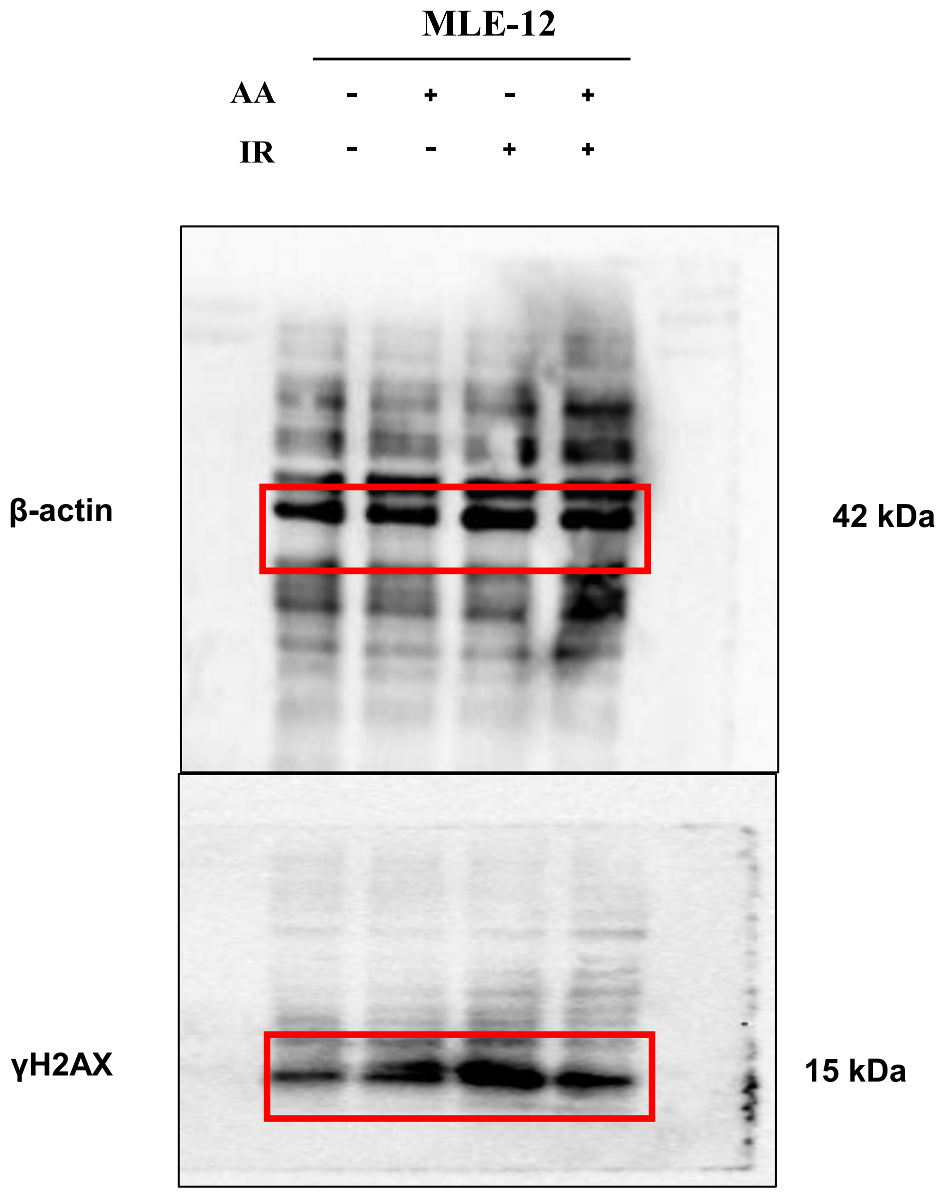

Figure 9E

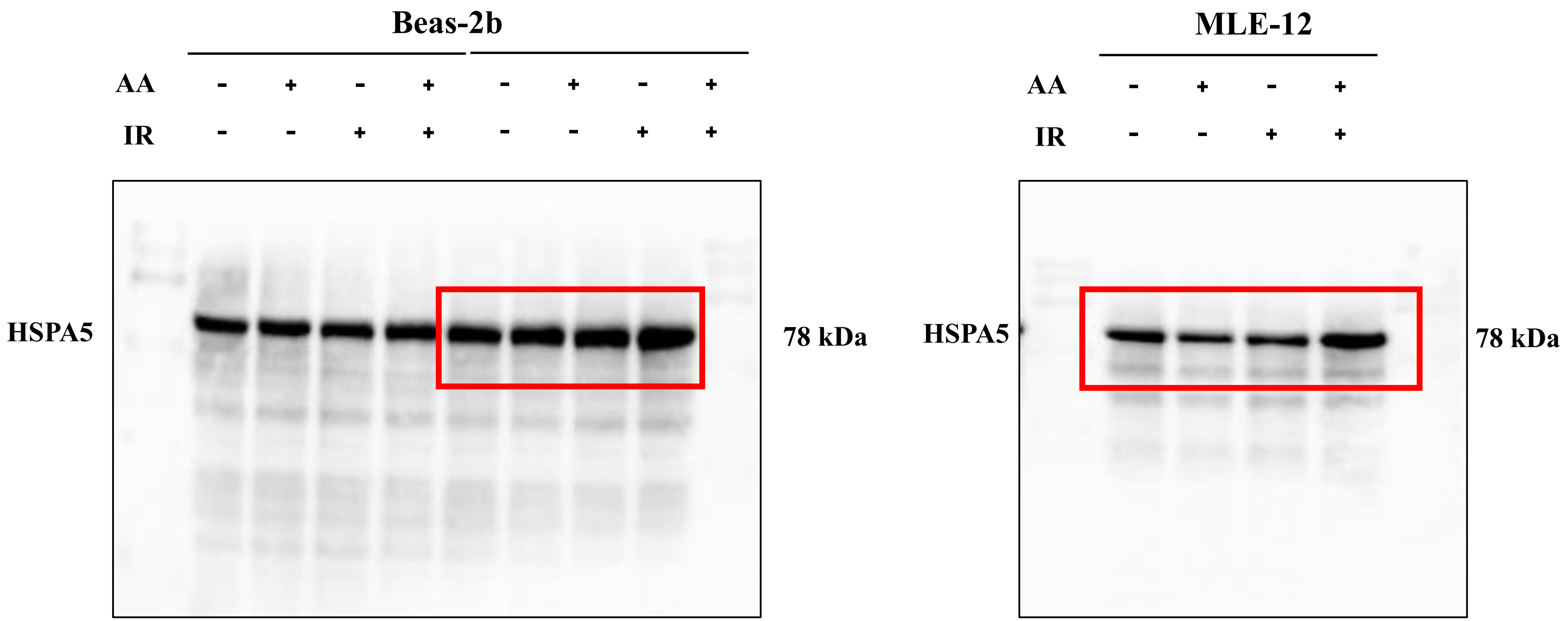

Figure 9E

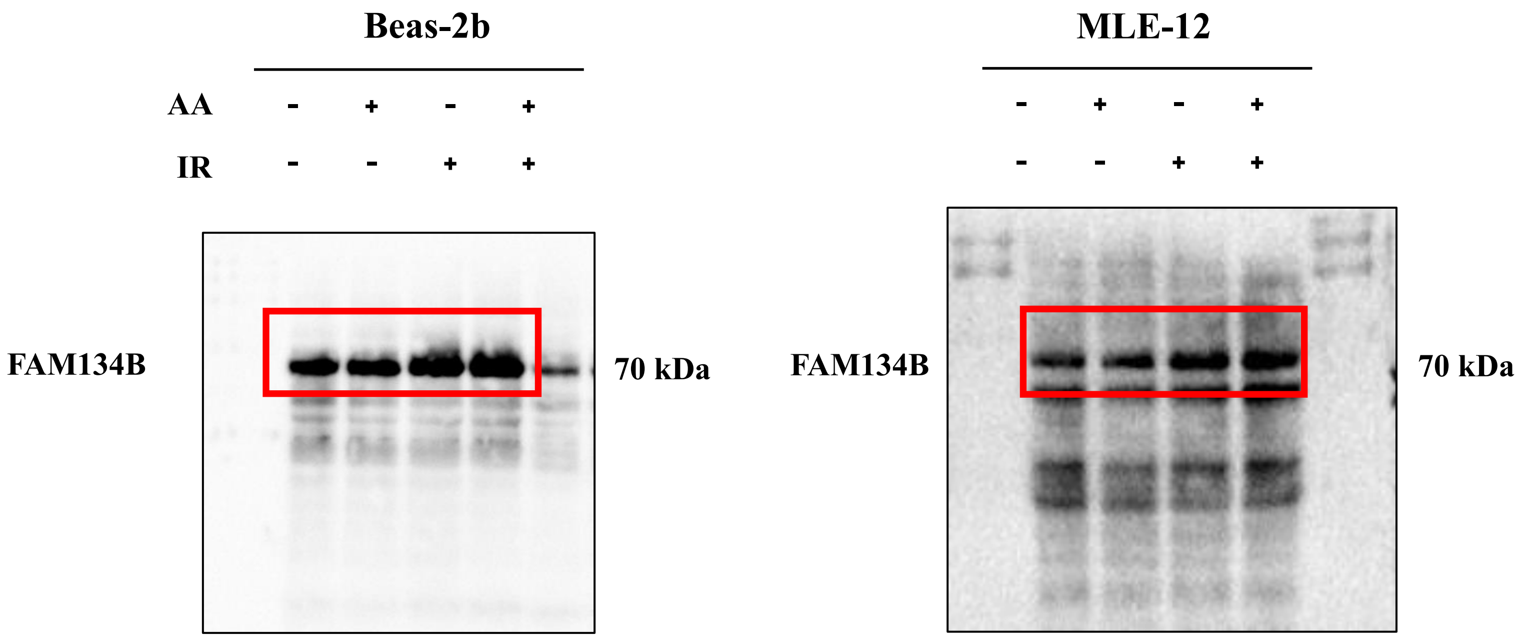

Figure 9E

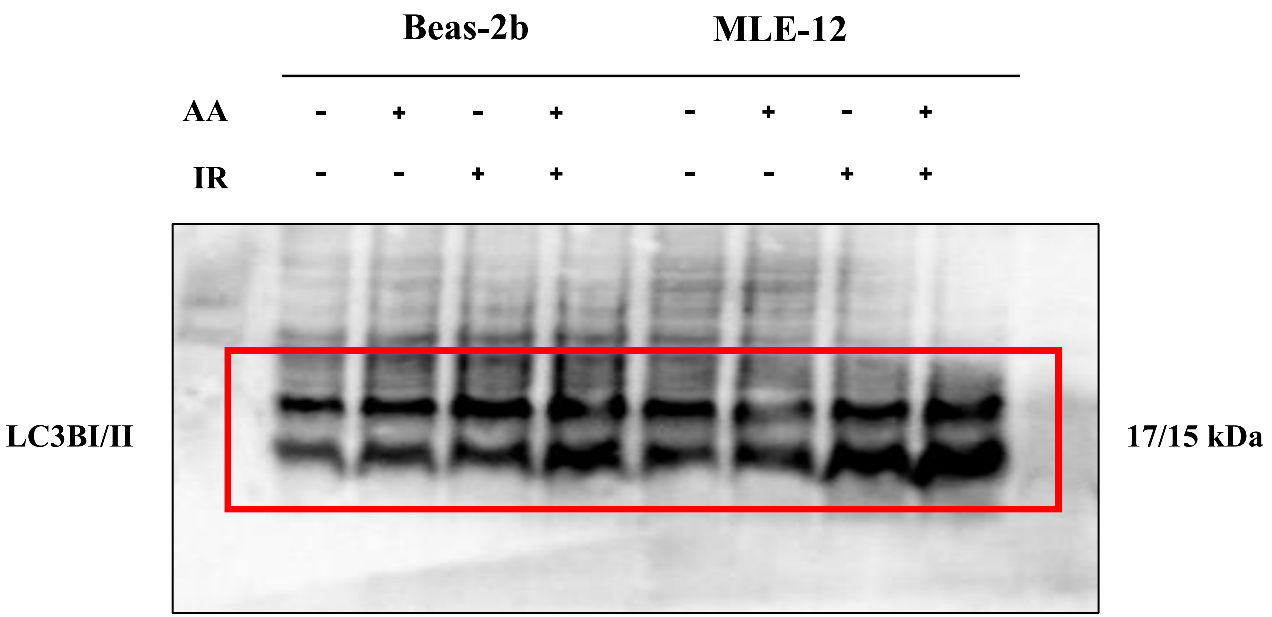

Figure 9E

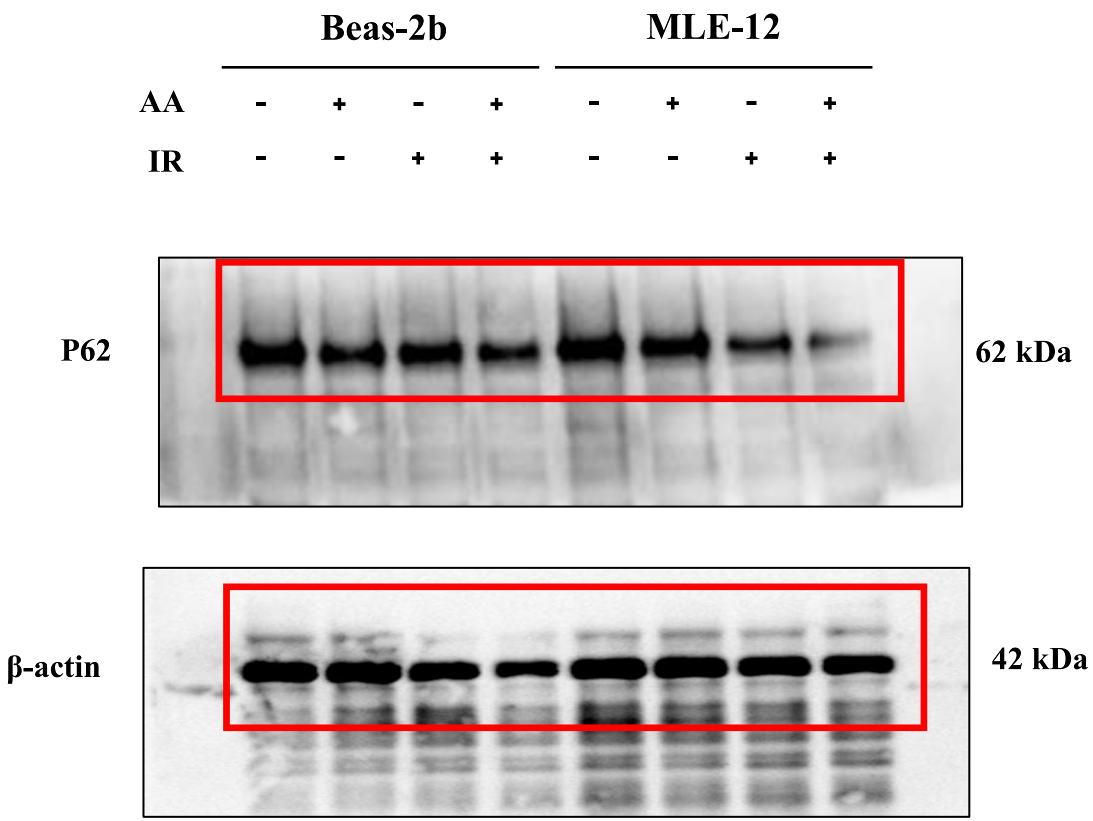

Figure 10A

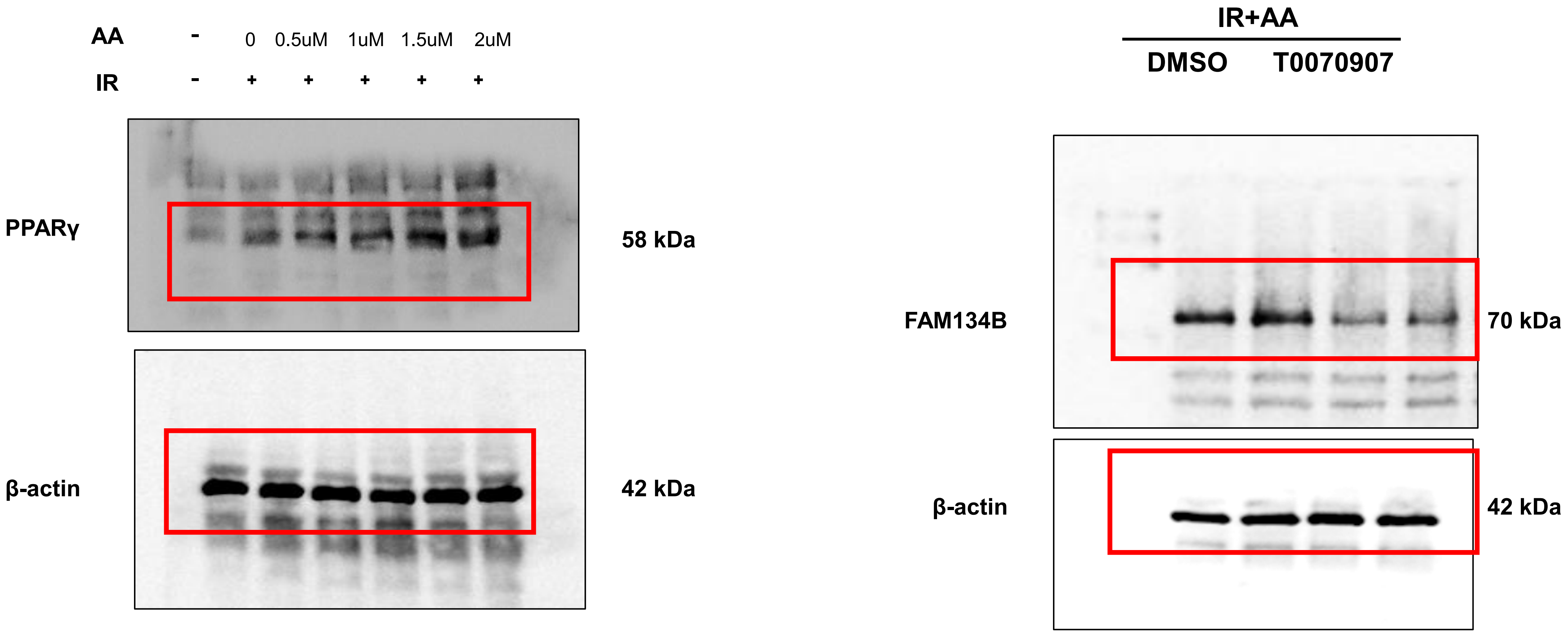
AA
-
0
0.5uM
1uM
1.5uM
2uM
IR
-
+
+
+
+
+
PPARγ
58 kDa
β-actin
42 kDa
IR+AA
DMSO
T0070907
FAM134B
70 kDa
β-actin
42 kDa

Figure 11K

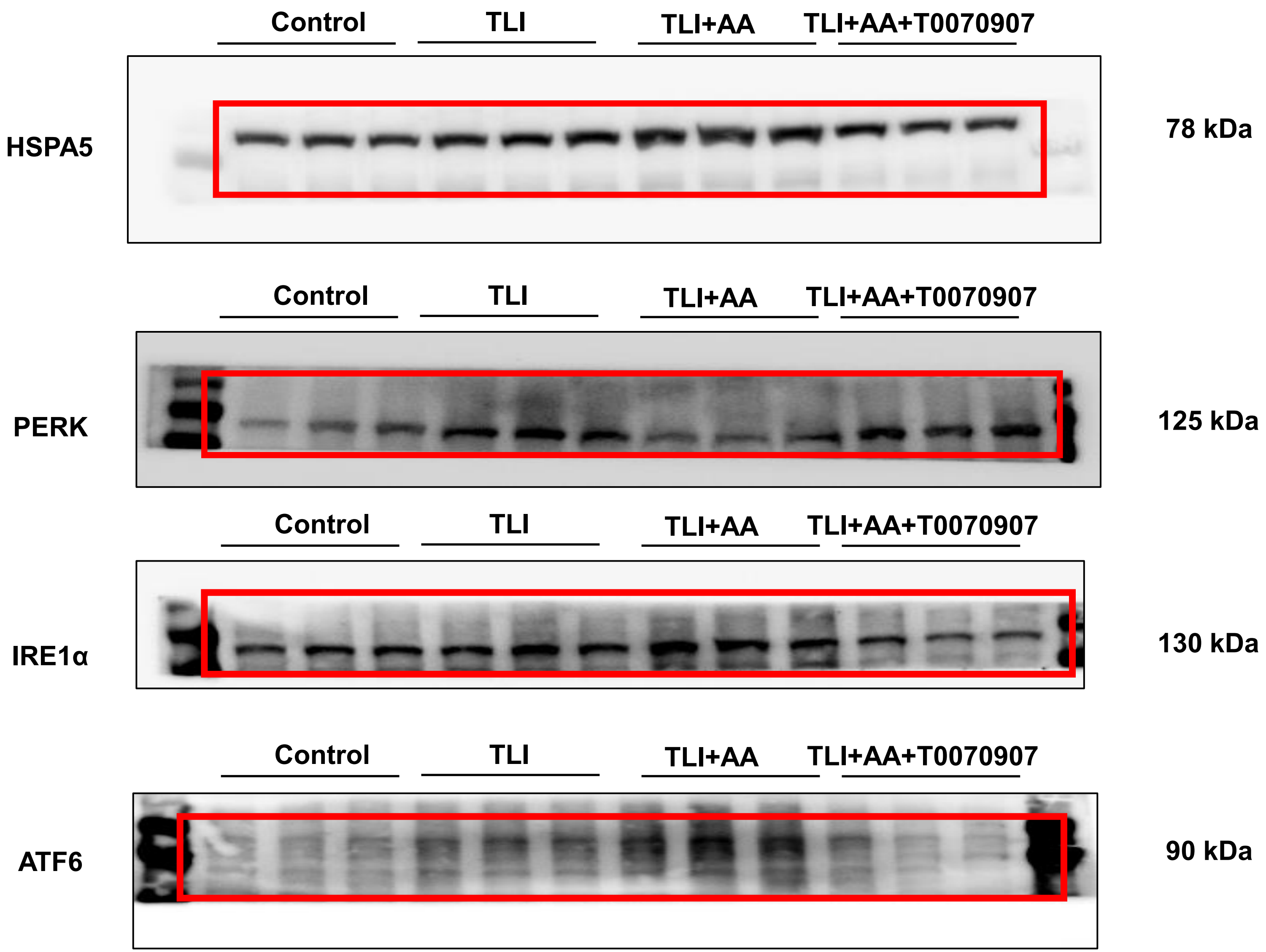

Control
TLI
TLI+AA
TLI+AA+T0070907
HSPA5
78 kDa
Control
TLI
TLI+AA
TLI+AA+T0070907
PERK
125 kDa
Control
TLI
TLI+AA
TLI+AA+T0070907
IRE1α
130 kDa
Control
TLI
TLI+AA
TLI+AA+T0070907
ATF6
90 kDa

Figure 11K

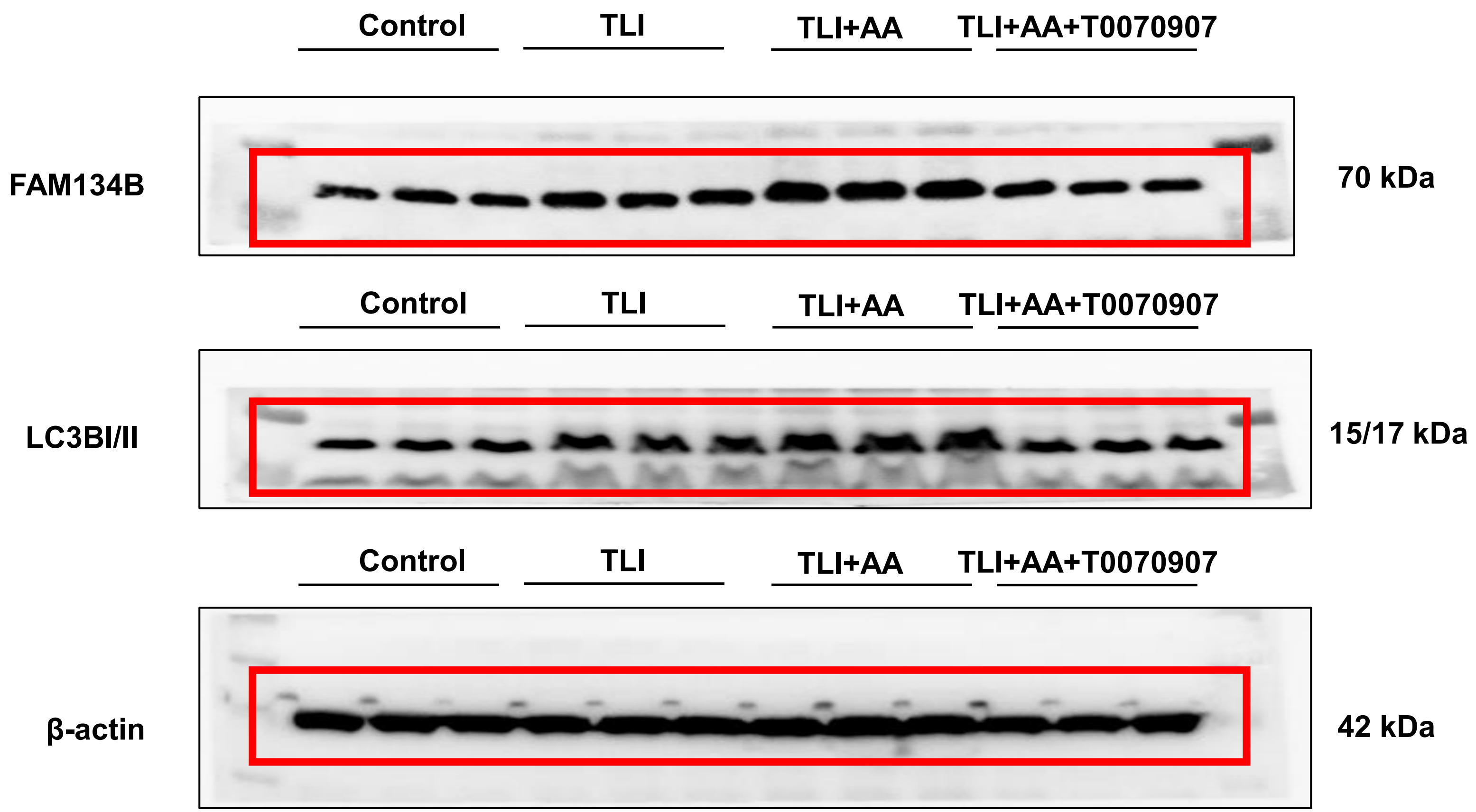
Control
TLI
TLI+AA
TLI+AA+T0070907
FAM134B
70 kDa
Control
TLI
TLI+AA
TLI+AA+T0070907
LC3BI/II
15/17 kDa
Control
TLI
TLI+AA
TLI+AA+T0070907
β-actin
42 kDa

Figure S3E

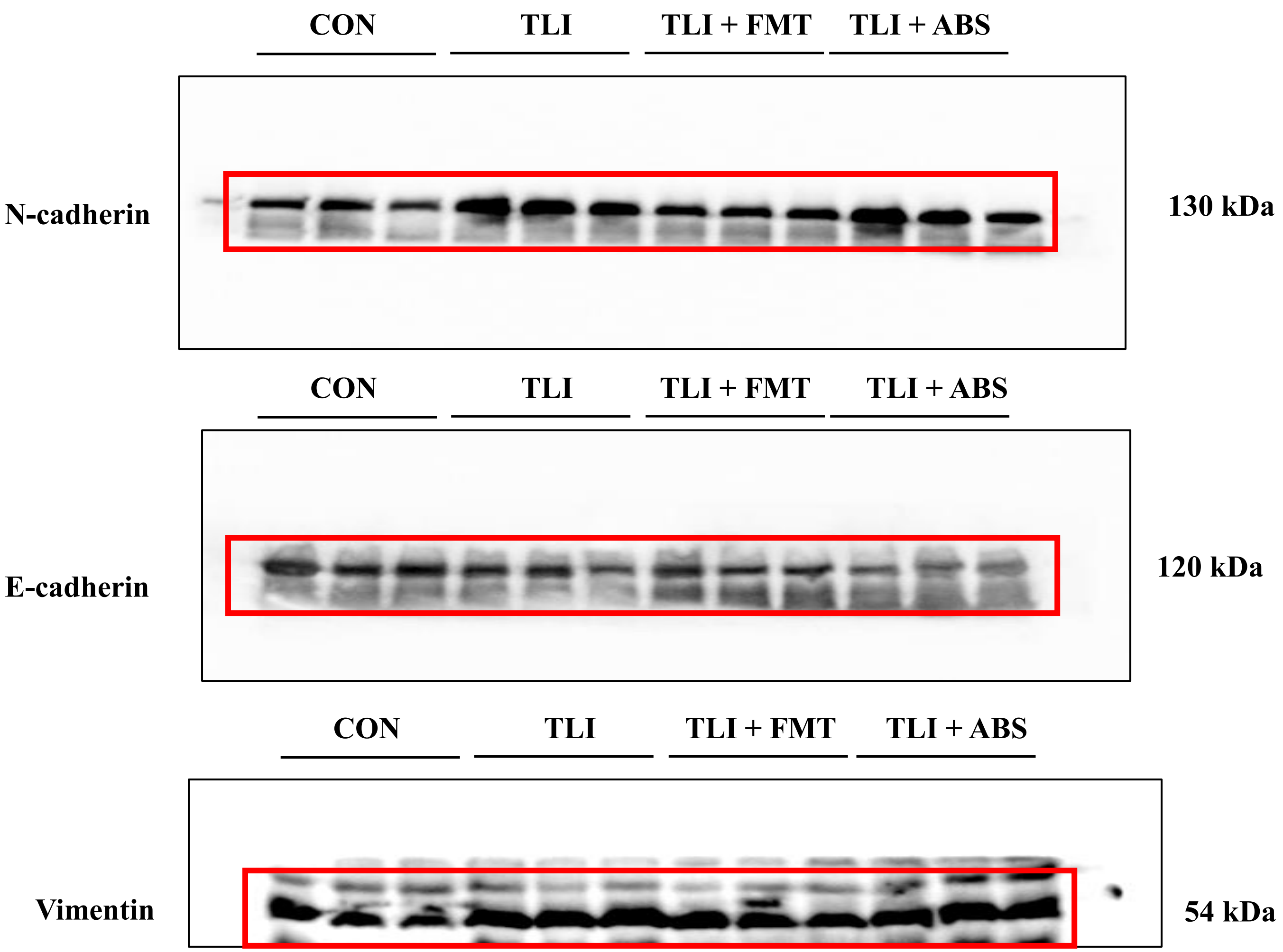
CON
TLI
TLI + FMT
TLI + ABS
N-cadherin
130 kDa
CON
TLI
TLI + FMT
TLI + ABS
E-cadherin
120 kDa
CON
TLI
TLI + FMT
TLI + ABS
Vimentin
54 kDa

Figure S3E

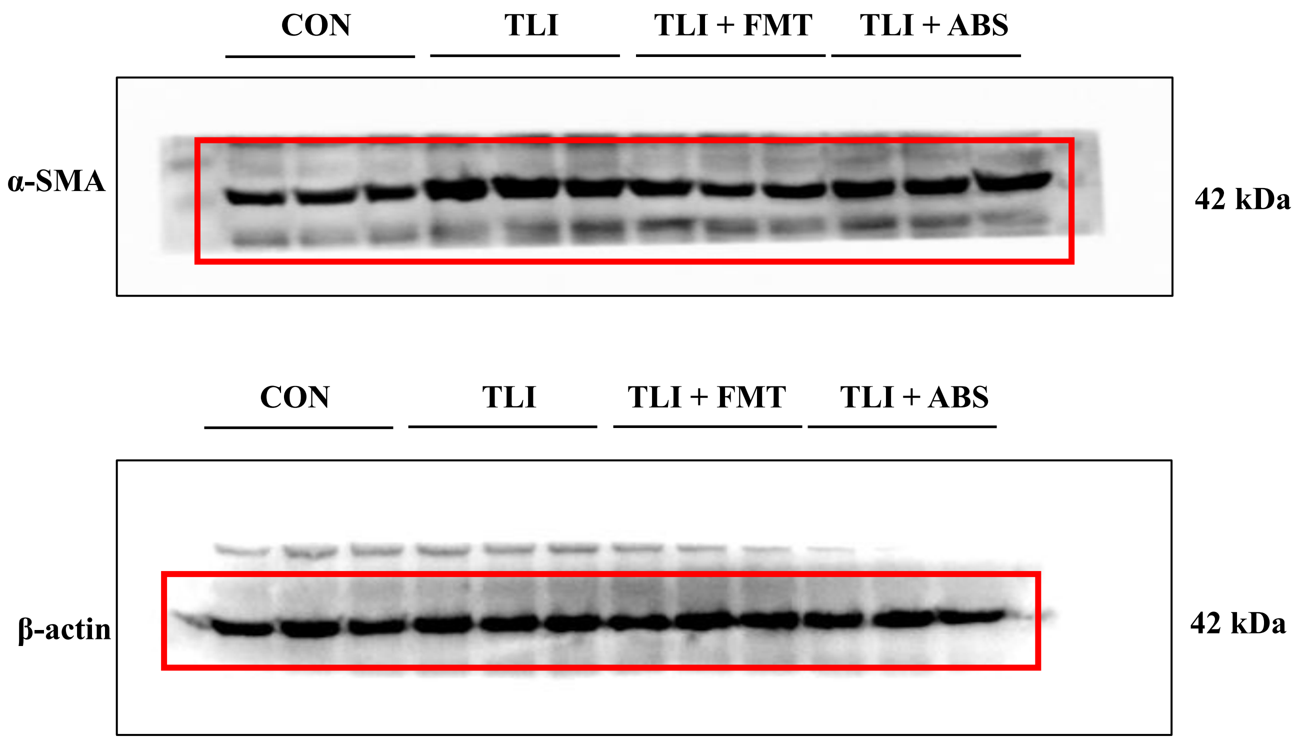
CON
TLI
TLI + FMT
TLI + ABS
α-SMA
42 kDa
CON
TLI
TLI + FMT
TLI + ABS
β-actin
42 kDa
